# Synthesis of magnetic electroactive nanomotors based on sodium alginate/chitosan and investigation the influence of the external electric field on the mechanism of locomotion

**DOI:** 10.1038/s41598-023-37463-9

**Published:** 2023-06-26

**Authors:** Fariba Mafakheri, Ali Asakereh, Sepideh Khoee, Mojtaba Kamankesh

**Affiliations:** Polymer Laboratory, School of Chemistry, College of Science, University of Tehran, Tehran, 14155-6455 Iran

**Keywords:** Materials for devices, Nanoscale materials

## Abstract

In this paper, we report a novel electric-driven Janus nanomotor (JNMs) based on SPIONs nanoparticle decorated with chitosan (Cs) and sodium alginate (**Na**/Alg) using the Pickering emulsion method. The JNMs dispersed in aqueous media exhibit linear trajectories under DC electric field, and the driving force is attributed to the self-electro-osmotic mechanism and surface modifications. This study offers an approach to remotely control the motion modes of the JNMs, including start, stop, directional and programmable motion, which can be advantageous for various application scenarios. The diffusion coefficient and velocity of the JNMs were investigated through mean square displacement analysis for single particle of JNMs, both in distilled water and in the presence of different di and trivalent metal cations (Fe^3+^, Al^3+^, Ba^2+^, Ca^2+^ and Mg^2+^) as crosslinking agents, as well as monovalent salts (LiCl and KCl). The results revealed that the motion of JNMs was fastest in the presence of Fe^3+^ as crosslinker agent (about 7.2181 μm^2^/s) due to its higher charge than equimolar Na^+^ . Moreover, it was demonstrated that increasing the ionic strength led to relatively higher speeds of JNMs, as the solution polarity increased and, as a result, the driving force of electro-osmoesis enhanced.

## Introduction

Micro/nanomotors have attracted much interest in the last decade due to their potential applications in biomedical engineering and environmental remediation^1,2^. Fuel-based micro-/nanomachines usually offer autonomous propulsion by decomposition of external chemical fuels (e.g. H_2_O_2_ and N_2_H_4_)^3,4^, which hinders their applications in biologically relevant media. Therefore, recent developments have focused on various micro/nanomotors powered by biocompatible fuels^5,6^. Fuel-free synthetic micro-/nanomotors powered by external stimuli (e.g. light, magnetic, ultrasonic, or electric fields)^7–10^ represent another attractive solution for practical applications owing to their biocompatibility and sustainability. These external stimuli are safe tools for precisely actuating micro/nanomotors toward the target regions in the preassigned tasks^11^. However, insufficient energy and uncontrollable directions of motion remain the main challenges to their promising applications. Recently, increasing attention has been paid to solving these problems, and numerous kinds of research have been devoted to the design and development of efficient, precise, and controllable micro/nanomotors using various methods. Xu et al. introduced synthetic Pt-Au nanomotors with rod morphology that showed controlled movement under acoustic field as external stimuli^12^. This study showed that these micromotors demonstrate on-demand reversible assembly and disassembly behavior by applying and withdrawing the acoustic source. Li et al. developed a special dual-mode magneto − acoustic hybrid nanomotor with rapid, on-demand, and programmable speed and direction control. These fuel-free nanomotors demonstrated an instantaneous switching between their acoustic and magnetic modes. As a result, this nanomotor can rapidly reverse its moving direction within less than 50 ms upon switching the operation model^13^.

However, the difficulty in applying these artificial micro/nanomotors in the biomedical industry lies in their nondurable propulsion (long wavelength of acoustic waves) for body tissues. Light and electrically driven micro/nanomotors have received considerable attention, especially in biomedical and pharmaceutical fields, due to their fuel-free propulsion, remote controllability, and harmlessness to cells and tissues^14,15^. This strategy not only provides the power movement of micro/nanomotors in a noncontact manner but also is able to concentrate active micro/nanomotors at predefined locations. In another instance, a new driving mechanism was reported that allows more switchable motion control on the orientations, alignment speeds, and directions of semiconductor silicon nanowires in the presence of an external electric field with simple visible-light exposure. The nanowire can be toggled instantly between parallel and transverse alignment to the E-field as a result of the interaction between the in-phase (real-part) electric polarization of the particles and the electric field^16^. Wu et al. reported the synthesis of self-propelled magnetic micromotors based on polystyrene spheres anisotropically coated with Cr/Ni/Au layers and investigated their propulsion under magnetic/electric hybrid power. They showed that the combination of a rotating magnetic field and an electric field could enhance micromotor mobility and direction control. In addition, this Janus microspheres could identify apoptotic cell among viable and necrotic cells based on their dielectrophoretic difference^17^. Xiao and coworkers reported fully fuel-free hybrid nanomotors that exhibited efficient propulsion in the presence of UV light and AC electric fields without adding any chemical fuel. They described bi-segmented hybrid Janus nanomotors in which both segments of TiO_2_ and Pt respond to UV and electrical fields. Since the two hemispheres of the TiO_2_-Pt micromotor polarize very differently under the electric field, the induced charge electroosmosis (ICEO) is produced with different magnitudes on the two surfaces, giving rise to an asymmetric flow that moves the particle forward. Both mechanisms, self-electrophoresis under UV light and induced-charge electrophoresis (ICEP) under AC electric fields, are well-documented and propel the particle towards the same direction, which resulted in up to 90% faster than simply adding up the speed powered by either source^18^.

Among the proposed micro/nanomotors powered by an electric field, polymer-based micro/nanomotors are of great significance for the ease of processing and facile functionalization, holding colossal potential for bioapplications^19^. Recent progress in this field was focused on metallodielectric patchy polymeric particles that enable active systems to perform sophisticated tasks in the future, such as selective cell treatment. Metallodielectric patchy particles are constructed from a metallic (gold) lobe and a dielectric (polymer) lobe. The charges responding to the AC field can propel the patchy particle under the ICEP mechanism with the dielectric lobe facing forward. These colloidal molecules exhibit controllable and switchable dynamic behaviors, such as propulsion, steering, and reconfiguration^20^. Recently has been shown that asymmetric metal patches on the surface of spherical colloids, in this case, polystyrene microspheres with metallic patches, can self-propel along non-linear helical trajectories powered remotely by an alternating current (AC) electric field by ICEP mechanism^21^.

In practice, electric motion can be generated by either the potential energy produced with ICEP and self-regulated electroosmosis from the DC or the AC electric field. ICEP is the most commonly used method to drive asymmetric micro/nanomotors with different shapes or polarizabilities in the electric field. The polarization of the asymmetric micro/nanomotors under an electric field induces an asymmetric accumulating countercharge in the electric double layer (EDL) around the motors and generates an electro-osmotic flow around these particles. The asymmetric EDL could be due to the different polarizabilities of the existing materials in the structure of micro/nanomotors. The more polarizable part of the EDL would accumulate more charges and form a stronger induced charge electro-osmosis (ICEO) to drive the asymmetric micro/nanomotors^22^. Practically, the motion control, especially acceleration or deceleration of velocity based on demand, can be directly influenced by tuning the strength of the electric field and the direction of movment is determined by the type of motors as well as the direction of the field^23^. Combining the electro-osmosis and the IECP caused by the asymmetric electrical polarization of the nanomotors under an electric field provides a strategy to synthesize electrically responsive nanomotors based on electroactive polymers^24^. For the first time, a new type of defective electric-driven micro/nanomotor with an asymmetric shape proposed, which can perform direction-switchable 2D motion behavior and bioinspired 3D helical motion behavior for cargo transportation. These nanomotors can be widely applied for various purposes, e.g. biomedicine, micro/nanosensors, and micro/nanomechanical systems. It is reported that the defective golden nanomotor can demonstrate controllable self-dielectrophoresis and ICEP motion behaviors in different frequency range through generating an asymmetric field gradient caused by structural deficiency^15^.

Herein, we synthesized fuel-free Janus micro/nanomotors (JNMs) powered by electrical fields. The JNMs were fabricated using Pickering emulsion consisting of a magnetite core (Fe_3_O_4_ NPs) anisotropically coated with chitosan (CS) and sodium alginate (**Na**/Alg). In This system, as an innocuous approach that is easy to operate, electric field has been used to produce the requisite propulsion. When the electric field is applied, the existing positive ions go toward the cathode, and the negative charge gathered around the sodium alginate polymer causes electroosmotic pressure, which causes the movement of these nanoparticles toward the opposite electrode. These micro/nanomotors exhibited a rapid oscillatory motion between the two electrodes which every single particle is propelled toward the anode electrode by a electro-osmotic mechanism. One of the intriguing advantages of these micro/nanomotors is that a controllable manipulator could be used to freely and easily start and stop the power source, allowing a switchable mode of movement of the micro/nanomotors in the presence or absence of an electrical field. Furthermore, such micro/nanomotors can be programmed to move along the intended trajectory in a controllable manner by adjusting the direction of the anode and cathode electrodes.

## Methods

### Materials and instruments

Ammonium hydroxide solution (25% NH_3_ in H_2_O), paraffin wax, 3-aminopropyl triethoxysilane (APTES), triethylamine (TEA), acryloyl chloride (AC), cisplatin, hydrogen peroxide 30% in water, tetrabutylammonium hydroxide (TBAOH), hydrochloric acid (HCl), fluorescein, N-Ethyl-N′-(3-dimethylaminopropyl)carbodiimide hydrochloride (EDC), Nhydroxysuccinimide (NHS), N,N-dimethyl sulfoxide (DMSO) and alginic acid sodium salt from brown algae with molecular weight 280,000 g/mol (determined by aq-GPC) were purchased from Merck chemical Co. Iron (II) chloride tetrahydrate, iron (III) chloride hexahydrate and sodium carbonate (Na_2_CO_3_) were obtained from Sigma-Aldrich. Chitosan ($\overline{{M }_{n}}$ = 3000 g/mol) was purchased from Golden-Shell Pharmaceutical Co. Ltd., China. Ethanol and chloroform were supplied from Kian Kaveh, Iran.

### Synthesis of acrylated sodium alginate

#### Synthesis of low molecular weight alginate

Degradation of high molecular weight sodium alginate to low molecular weight sodium alginate was prepared using a modified procedure based on previous reports^25^. A 1000 mL round bottom flask was charged with 7.5 g of high molecular weight sodium alginate in 500 mL of distilled water (1.5% w/v). Then 3.5 g of ascorbic acid and 5 mL of pyridine were added to the reaction medium, and the mixture was stirred at room temperature for 15 min. The flask was fitted with a rubber septum and placed under Ar atmosphere; the resulting solution was placed in an oil bath and heated up to 80 °C. Followed by adding 50 mL of 30% hydrogen peroxide to the reaction via syringe, the reaction mixture was then stirred at 80 °C for 2 hrs. The low molecular weight sodium alginate powder was obtained by lyophilizing from water and analyzed by aqueous gel permeation chromatography (aq-GPC).

#### Synthesis of tetrabutylammonium alginate (TBA/Alg)

In the next step, for better dissolution of the polymer in non-aqueous solvents for acrylation, cation exchange with tetrabutylammonium hydroxide (TBAOH) was performed using previous methods^25^. 250 mL round bottom flask was charged with 100 mL solution of ethanol/0.6 M HCl in water (1:1 v/v), then dried low molecular weight sodium alginate (1 g) was added to the solution. The flask was fitted with a rubber septum and the mixture solution was stirred at 4 °C for 24 hrs. The product was filtered by vacuum and purified by recrystallization in ethanol and then concentrated under reduced pressure to produce low molecular weight alginic acid. A dilute solution (4% w/v) of tetrabutylammonium hydroxide (TBAOH) was added dropwise to the solution of low molecular weight alginic acid in water (3% w/v), until the pH value was adjusted between 7 and 9 by controlling with pH-meter. The reaction mixture was stirred at room temperature for 24 hrs. The final product (**TBA**/Alg) was precipitated in ice-cold ethanol, centrifuged and washed with ethanol (3 times). The dry product was characterized by 1H-NMR in D_2_O^25^.

#### Synthesis of acrylayed sodium alginate (AcNa/Alg)

Acrylayed sodium alginate (Ac**Na**/Alg) was prepared according to Vitaliy and Ronald procedure with slight modifications^25^. Briefly, the synthesized **TBA**/Alg (666 mg, 4.16× 10^–2^ mmol) and triethylamine (1.11 mL, 8 mmol) were dissolved in 6 mL DMSO with 1% tetrabutylammonium fluride (TBAF) at 0 °C. Acryloyl chloride (0.48 mL, 6 mmol) was then added dropwise to the mixture, and the stirring was continued at room temperature for 48 hrs, under a dry nitrogen atmosphere. Afterward, the acrylated tetrabutylammonium alginate (Ac **TBA**/Alg) was obtained by pouring the reaction solution into cold water and then filtering the precipitate. By adding sodium carbonate solution to the suspension of Ac**TBA**/Alg in water, the water-soluble acrylated sodium alginate (Ac**Na**/Alg) was produced, which was isolated by precipitation in ice-cold ethanol, washed with water, and then lyophilized from water. Ac**Na**/Alg was dried in a vacuum oven at 40 °C overnight for further characterization with 1HNMR (in D_2_O).

### Synthesis of Cs-Fe_3_O_4_-Na/Alg Janus nanoparticles

#### Synthesis of magnetite nanoparticles half-coated with chitosan

The method reported in a previous work^26^ (S1.1-S1.6) was used to synthesize Cs-cisplatin-Fe_3_O_4_ Janus nanoparticles. To decorate the half-part of the NPs with hydrophilic groups, half of the prepared SPION was first entrapped in wax droplets using the Pickering emulsion method, and then the remaining bare surfaces were modified with APTES. In the next step, the attachment of chitosan to the bare surface of SPIONs was required, for which cisplatin was selected as a linker to conjugate chitosan to the surface of SPIONs. The reaction between the chloride ligand of cisplatin and the amine groups of APTES via nucleophilic substitution was used to react cisplatin with the remained amine groups of APTES. To achieve chitosan-coated Janus NPs, all of the half-masked NPs (Cisplatin -APTES-Fe_3_O_4_-wax microspheres) obtained in the preceding step were dispersed in deionized water and mixed with chitosan. The amino functional group of the chitosan replaced the chlorine group functionalized particles via the ligand exchange method. In the next step, the chitosan-Cisplatin APTES-Fe_3_O_4_/wax droplets obtained were filtered and washed with deionized water to remove free particles and excess chitosan. All these procedures were performed at room temperature. Finally, the unmasking Cs-cisplatin-Fe_3_O_4_ Janus nanoparticles (JNPs) were collected after washing them with chloroform at 70 °C using the bain-marie technique.

#### Functionalization of the bare side of Cs-cisplatin-Fe_3_O_4_ JNPs with 3-aminopropyl triethoxysilane (APTES)

Cs-cisplatin-Fe_3_O_4_ nanoparticles (300 mg) were dispersed in 5 mL ethanol then APTES (0.46 mL, 2 mmol) and distilled water (0. 5 mL) were added to the dispersion, and the mixture was kept stirring at room temperature for 24 h. The resulting mixture was washed three times with ethanol to remove the unreacted amount of APTES, and the amine-decorated Cscisplatin-Fe_3_O_4_ nanoparticles (Cs-cisplatin-Fe_3_O_4_-APTES) were kept in distilled water for further reactions.

#### Synthesis of Janus (Cs-Fe_3_O_4_-Na/Alg) nanoparticles

As the first step, 2g (0.125 mmol) acrylated sodium alginate (Ac**Na**/Alg) was dissolved in distilled water (70 mL) and the solution was stirred at room temperature for 30 min (solution 1). Cs-cisplatin-Fe_3_O_4_-APTES (0.5 g) was dispersed in distilled water (5 mL) under ultrasonic irradiation for 10 min and rapidly added to the solution 1, and kept stirring at room temperature for 72h. The obtained Janus nanoparticles coded as (Cs-Fe_3_O_4_-**Na**/Alg) JNPs were separated using external magnetic field and were washed five times with distilled water and kept in distilled water.

### Synthesis of fluorescently labeled Janus (Cs-Fe_3_O_4_-Na/Alg) nanoparticles

#### Synthesis of fluorescein-labeled cisplatin (FL-CisPt)

33.23 mg (0.1 mmol) fluorescein was added to 4 mL distilled water and allowed to dissolve completely in water. Then 11.509 mg (0.1 mmol) NHS was added to the reaction mixture at room temperature and stirred for 5 min. Followed by the addition of 28.755 mg (0.15 mmol) EDC in 2 mL H_2_O, the reaction mixture was stirred at room temperature for 4 h, then (0.1 mmol) cisplatin was added to the mixture and the reaction media was stirred at room temperature for 24 h. After completing the reaction the product was extracted with DCM (5×60mL). The combined organic phases were dried over Na_2_SO_4_ and concentrated under vacuum to produce a yellow viscous liquid with a 55% yield. For fluorescence imaging, the dye-tagged cispaltin was dissolved in dichloromethane and then dried on the glass slides.

#### Synthesis of fluorescein-labeled APTES-Fe_3_O_4_ (FL-APTES-Fe_3_O_4_) JNPs

144.70 mg (0.25 mmol) of fluorescently labeled Cisplatin (FL-CisPt) was dissolved in 2 mL dichloromethane and was added to 40 mg of wax-free APTES-Fe_3_O_4_ JNPs dispersed in 3 mL dichloromethane and let to stir for 72 h. The product was collected and stored in ethyl alcohol. For fluorescence imaging, FL-APTES-Fe_3_O_4_ JNPs were dispersed in ethanol and then dried on the glass slides.

#### Synthesis of fluorescein-labeled Cs-cisplatin-Fe_3_O_4_ (FL-Cs-cisplatin-Fe_3_O_4_) JNPs

Fluorescein (10 mg, 0.03 mmol) was dissolved in 1 mL of water and added to a dispersion of wax-free Cs-cisplatin-Fe_3_O_4_ JNPs (20 mg) in water (3 mL). Followed by the addition of 0.93 mg (4.8 × 10^−3^ mmol) of EDC and 0.69 mg (6 × 10^−3^ mmol) of NHS, the mixture of nanoparticles stirred under a stirring rate of 600 rpm at room temperature for 24 h. Prepared FL-Cs-cisplatin-Fe_3_O_4_JNPs were then collected using an external magnetic field and washed with pure distilled water to remove the excess amount of fluorescein. The product was kept in ethyl alcohol in the dark. For fluorescence imaging, FL-Cs-cisplatin-Fe_3_O_4_ JNPs were dispersed in ethanol and then dried on the glass slides in the dark.

### Characterization

^1^H NMR spectra were taken by Bruker DRX 500 spectrometer (500 MHz, Germany) in D_2_O. IR evaluations were recorded on Bruker EQUINOX 55 model FTIR. The attachment of cisplatin to APTES-Fe_3_O_4_-wax microspheres was evaluated with Energy Dispersive X-Ray Analysis (EDX) (Bruker X-Flash spectrometer). The prepared Janus nanoparticles were characterized by scanning electron microscopy (SEM) (HITACHI S 4160). The samples were placed on a stub and sputter-coated with gold before observation. The size and shape of nanoparticles can be obtained from these images. The percentage of attached polymers on the surface of the nanoparticles was determined by Thermal gravimetric analysis (TGA) (TGA Q50 V6.3 Build 189) in a temperature range between 25 and 600 °C at a rate of 10 °C /min under nitrogen atmosphere. The nanoparticle size distribution and zeta potential of Fe_3_O_4_ NPs, APTES-Fe_3_O_4_, cisplatin-Fe_3_O_4_, Cs-cisplatin-Fe_3_O_4_ and Cs-Fe_3_O_4_-**Na**/Alg JNPs was determined using Dynamic Light Scattering (DLS) (Nano ZS, Malvern instrument, UK). Before measurement, the nanoparticles were sonicated for 10 min to prepare a homogeneous solution and finally, the size distribution of nanoparticles was measured at 25 °C. Zeta potential is an important index of surface charge which represents particle stability in dispersion. As mentioned above, after dispersing and sonicating the nanoparticles, zeta potential analysis was performed by DLS. The dried size and morphology of Janus Cs-Fe_3_O_4_-**Na**/Alg nanoparticles were measured using transmission electron (TEM) (Tecnai G2 F20 STwin). The nanoparticles were dispersed in deionized water and a drop of nanoparticles suspension was placed on a carbon-coated grid. The grid was completely dried and used for TEM analysis (150 kV). The weight average molecular weight ($\overline{{M }_{w}}$), number average molecular weight ($\overline{{M }_{n}}$), and polydispersity index (PDI) of sodium alginate after degradation were determined by using the gel permeation chromatography (GPC) (PL-EMD 950). The motion behavior of Cs-Fe_3_O_4_-**Na**/Alg JNMs were recorded using a Motic AE 31 optical microscope by applying an electric field in distilled and deionized water media. The movement of the nanomotor is toward the anode electrode; therefore, the direction of the motion will be reversed by switching the anode and cathode. Electrical control of the movement of micromotors is done by applying "on" and "off" electric current with different time intervals and recording with a camera. It is necessary to mention that the length of time of being "off" and "on" is identical, and it is equal to the number that is expressed by the word "pulse" in videos. As well as, a transient change in the amplitude of the electric field from the baseline 0 V to 1.5 V and 3Vwas applied and the JNMs movements were recorded. Next, the effect of crosslinking ions on the physical properties and velocity of JNMs was evaluated by adding an equimolar solution of divalent and trivalent metal cations (BaCl_2_, CaCl_2_, MgCl_2_, FeCl_3_ or Al_2_(SO_4_)_3_) to the pristine JNMs and their motions were recorded under an electric field with an amplitude of 1.5 V. For this purpose, a solution of each salt with a concentration of 1 mmol in 10 mL distilled water was prepared. Next, each solution was transferred to a beaker containing a dispersion of 5 mg Cs-Fe_3_O_4_-Na/Alg in 10 mL distilled water, and the mixture was stirred for 30 min. The highly diluted solutions were prepared by adding a drop of every solution into 9 mL of distilled water to acquire high-quality trajectories. The framerate of all videos was 66 frames per second and the magnification of the microscope was 400x. After that videos were magnified 4 times for more reliable tracking.

## Results and discussion

### Synthesis and characterization of magnetite-based chitosan/sodium alginate JNPs

**Figure 1:**
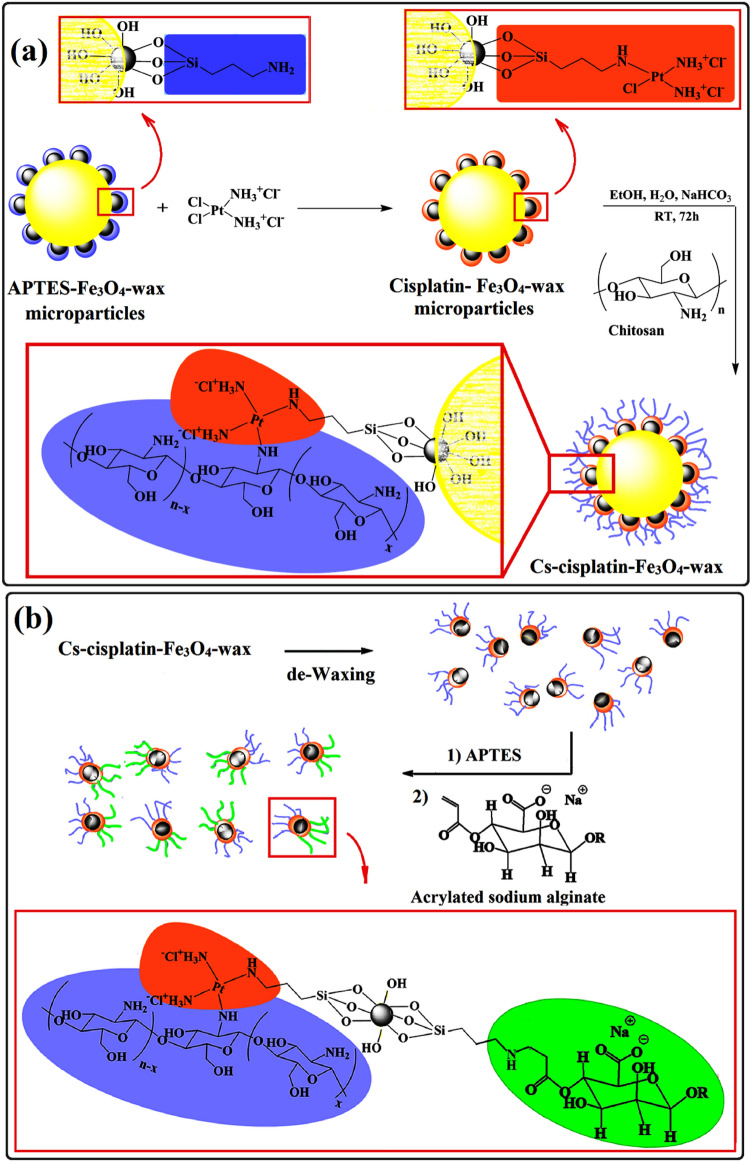
Summary of synthetic route for preparation of: (**a**) Cs-cisplatin-Fe_3_O_4_-wax micrparticles and (**b**) Chitosan/sodium alginate Janus nanoparticles (Cs-Fe_3_O_4_-**Na**/Alg JNPs) (This figure was created by Paint: https://apps.microsoft.com/store/detail/paint/9PCFS5B6T72H version: 11.2302.18.0).

New technologies are being developed to improve targeted drug delivery using catalytic nano/micromotors. By modifying their structures, performance can be enhanced and adverse effects minimized. This paper focuses on developing a new manufacturing method for swimming devices with two key features: (i) the ability to move without chemical fuel; and (ii) directional control through a biocompatible polymer coating. The aim is to create a Janus nanomotor using biodegradable polymers like sodium alginate (**Na**/Alg) and chitosan (Cs) by the Pickering emulsion method, that moves with an electrical field and electro-osmosis mechanism. These motors offer a safer option for biomedical applications such as drug delivery, micro-manipulation, and bio-imaging-guided procedures. For this purpose, SPION nanoparticles were synthesized and trapped in wax microspheres according to Fig. S1, **Step 1**^26^. The non-coated faces of nanoparticles were modified with APTES (Fig. S1, **Step 2**)^26^. APTES-Fe_3_O_4_ wax microspheres can be easily dispersed in water media due to the presence of amine functional groups on the half surface of the nanoparticles after the reaction of SPION with APTES. In the next step, our target is the chemically linking chitosan to the non-protected surface of SPION nanoparticles. For this purpose, cisplatin was selected as a linker and conjugated to the bare surface of modified SPIONs via the nucleophilic substitution reaction between the chloride ligand of cisplatin and the amino groups of nanoparticles (Fig. 1a). Energy-dispersive X-ray spectroscopy (EDX) was used to identify the chemical compositions of wax-free cisplatin-Fe_3_O_4_ Janus nanoparticles*.* For this purpose, a small amount of cisplatin-Fe_3_O_4_-wax microparticles were taken and de-waxed by chloroform to produce wax-free cisplatin-Fe_3_O_4_ Janus nanoparticles. The EDX spectrum approves the presence of C, Fe, O, Si, and Pt with a weight percent of 15.73, 35.41, 36.02, 5.52, and 7.33 wt.%, respectively, in nanoparticles (Fig. S2). To confirm the synthesis of the APTES-Fe_3_O_4_ JNPs and its functionalization with cisplatin, we used the FTIR technique. In the FTIR spectrum of magnetite nanoparticles (Fig. S3, blue line), the intense peak at 564 cm^–1^ is attributed to the stretching vibration mode associated with the metal–oxygen (Fe–O) bonds in the crystalline lattice of Fe_3_O_4_. A band at 1627 cm^–1^ and the broad band centered at 3400 cm^–1^ are related to the presence of hydroxyl groups and attributed to OH-bending and OH-stretching, respectively. Fig. S3, black line shows the FT-IR of half-coated APTES-Fe_3_O_4_ nanoparticles. The characteristic peak of this compound is Si–O–Si bonds that was found at 1030 cm^–1^, C–H stretching vibration of the propyl groups can be seen at 2852 and 2932 cm^−1^. The N–H bending and stretching vibration of terminal primary amine groups cannot be seen in this spectrum**,** due to the overlaping with the already existing magnetite peaks at 1627 and 3419 cm^−1^. The FT-IR spectrum of de-waxed cisplatin-Fe_3_O_4_ JNPs (Fig. S3, red line) was compared with APTES- Fe_3_O_4_ JNPs (Fig. S3, black line). The O–H and N–H pattern in the 3157 cm^–1^ region for nanoparticles before and after the reaction with cisplatin was more or less similar. The cisplatin-reacted nanoparticles display the series peaks related to tensional trembles of ammonium groups of cisplatin moieties at 1228, 1303, and 1643 cm^−1^^27^.

Fluorescein was incorporated into the Fe_3_O_4_-APTES JNPs shell to verify the carried-out reactions. For this purpose, cisplatin was pretreated with fluorescein (FL-CisPt) (Fig. S4) and subsequently reacted with amine groups already available on the Fe_3_O_4_ surface after the APTES reaction (Fig. S5). Figure S4 illustrates the fluorescence microscopy images of cisplatin before and after incubation with fluorescein. Cisplatin aggregates are apparent in their pure form, while, after treatment with fluorescein, a good dispersion of the FL-CisPt sample discolored to the green color is observable. The formation of the FL-CisPt was confirmed by UV absorption spectra analysis and compared with the pristine cisplatin and fluorescein (Fig. S6). The characteristic absorption peaks of FL-CisPt at 207 and 494 nm correspond to the fluorescein and platinum segments, respectively, with a slight shift. In the next step, fluorescein-tagged and -untagged cisplatin were reacted with APTES-Fe_3_O_4_ JNPs (Fig. S5), and their connection was evaluated by Fluorescence spectroscopy. The fluorescent color observable in the fluorescein-tagged sample proved the tagging reaction. Due to the presence of SPION in the fluorescein-tagged SPION compound, its characterization by UV spectroscopy is impossible.

The main purpose of using cisplatin in this work was to bind chitosan to the half-surface of SPION. Accordingly, chitosan amine groups attached to the cisplatin remained chloride groups via the ligand exchange method (Fig. 1a). The mean diameter of Cs-CisPt-Fe_3_O_4_-wax microparticles was determined using optical microscopy and Image J software. Analysis of 100 particles yielded a mean diameter of 4.55 ± 1.43 µm (Fig. S7). Cs-cisplatin-Fe_3_O_4_-wax microparticles were washed off with chloroform to remove the wax, dry in the oven at 40 °C, and then analyzed by FT-IR spectroscopy to characterize the produced chitosan half-coated nanoparticles. The typical FT-IR spectra of pure chitosan and Cs-cisplatin-Fe_3_O_4_ JNPs are shown in Fig. S8. The peak at 3300 to 3500 cm^−1^ was related to the stretching vibrations of chitosan hydroxyl groups. The bsorption peaks at 1630 and 1531 cm^−1^ are due to the N–H bending vibration of protonated amine (−NH_2_) groups and C–H bending vibration of the alkyl groups, respectively. The anti-symmetric stretching vibration of C–O–C bridges and glucopyranose ring of chitosan were located at 1059 and 886 cm^−1^, respectively. Cs-cisplatin-Fe_3_O_4_ JNPs demonstrate the characteristic peaks of cisplatin at 699 and 756 cm^−1^, as well as all the chemical bonds of pure chitosan, but with small displacements. This displacement indicates the chemical reaction between cisplatin found at the half-surface of modified nanoparticles and chitosan. Fluorescein was reacted with chitosan of the Cs-cisplatin-Fe_3_O_4_ JNPs via EDC/NHS chemistry to prove the presence of chitosan in half of the structure of Janus nanoparticle. The fluorescein-tagged nanoparticles exhibited a bright green light emission of the fluorescein segment at one-half of the nanoparticles under a UV lamp (Fig. S9).

**Figure 2:**
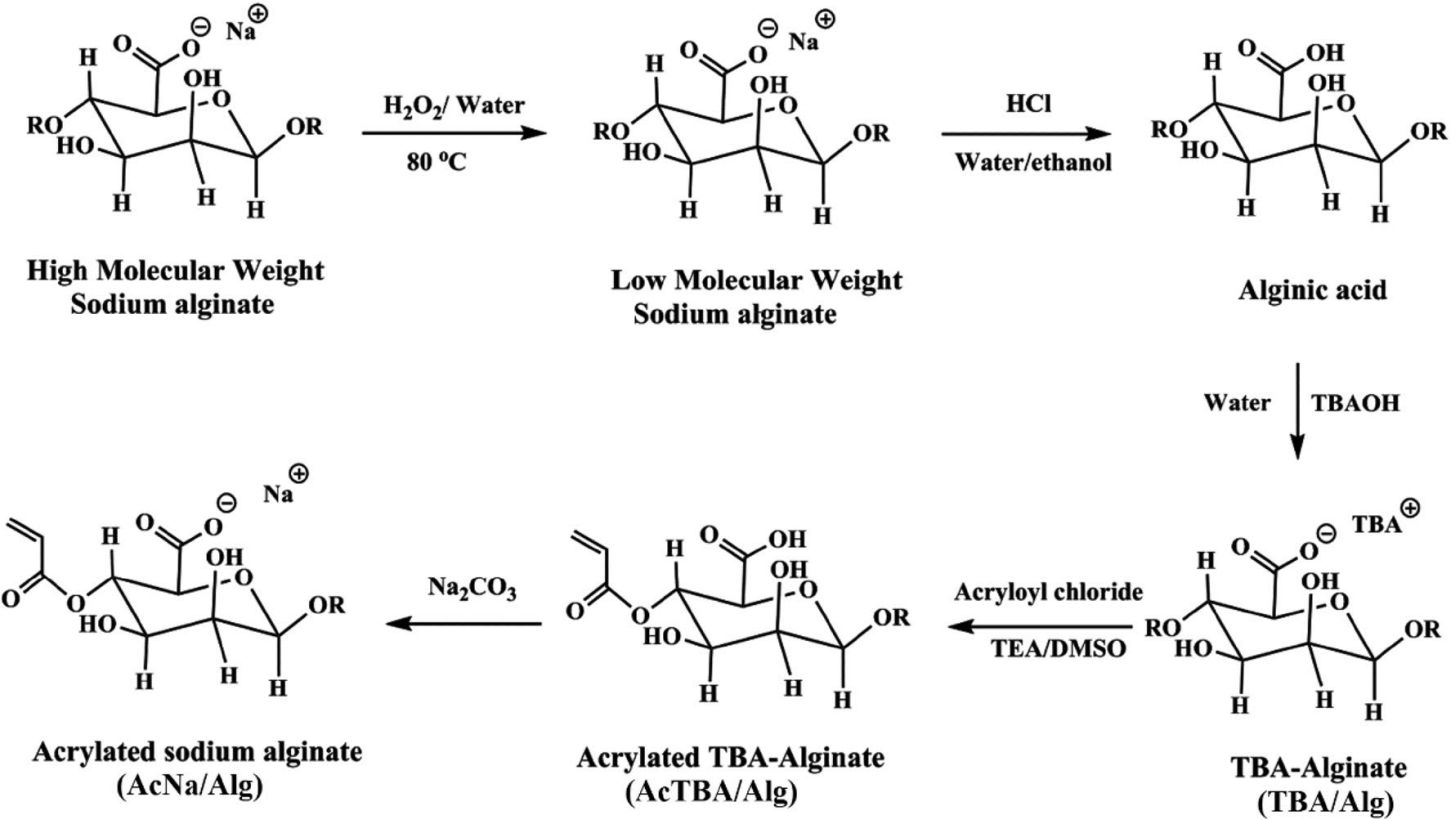
Schematic of synthetic route for preparation of alginate which shows one of four possible ways acryloyl chloride could be esterified onto alginate backbone.

Till here, half of the surface of magnetite nanoparticles was covered by chitosan. For the synthesis of Janus nanoparticles containing chitosan and alginate domains, the wax removal from the Cs-cisplatin-Fe_3_O_4_-wax microparticles was performed by chloroform. Then by the reaction of Cs-cisplatin-Fe_3_O_4_ NPs with acrylated alginate, the final CsFe_3_O_4_-Alg JNPs were prepared as shown in Fig. 1b. The total synthesis of acrylayed sodium alginate (Ac**Na**/Alg) has been achieved in five steps, as illustrated in Fig. 2.

Due to the low solubility of pristine alginate in organic solvents, at first, high molecular weight alginate was depolymerized into shorter fragments using hydrogen peroxide (H_2_O_2_) at 80 °C. In the next step, Alginate-TBA was synthesized through the cation exchange reaction via the reaction of the LMW-alginic acid with tetra butyl ammonium hydroxide (TBA-OH) to improve its solubility in DMSO. Modification of TBA Alginate occurred when hydroxyl groups of alginate reacted with acryloyl chloride in DMSO and produced acrylated TBA alginate (Ac**TBA**/Alg). Neutralization with sodium carbonate resulted in the formation of acrylated sodium alginate. Measuring molecular weight after depolymerization reaction (i.e. 1st step) using aq-GPC showed that weight average molecular weight (MW) of the polymer was 24,000 g/mol with polydispersity index (PDI) of 2.23. The product of LMW-sodium alginate with HCl obtained from the second step was characterized by FT-IR spectroscopy. As illustrated in Fig. S10, the stretching vibrations of asymmetric and symmetric bands of carboxylate anions are located at 1620, 1432, and 1300 cm^−1^, respectively, and the peak appeared at 3400 cm^−1^ corresponds to stretching vibrations of hydroxyl groups. The formation of alginic acid was confirmed by the shift of the characteristic carbonyl group peak from 1620 to 1726.08 cm^−1^, which is due to the conversion of sodium alginate carboxylate groups to alginic acid carboxylic acid groups. The positions of the other peaks are nearly identical for the two above-mentioned alginate derivatives.

**Figure 3:**
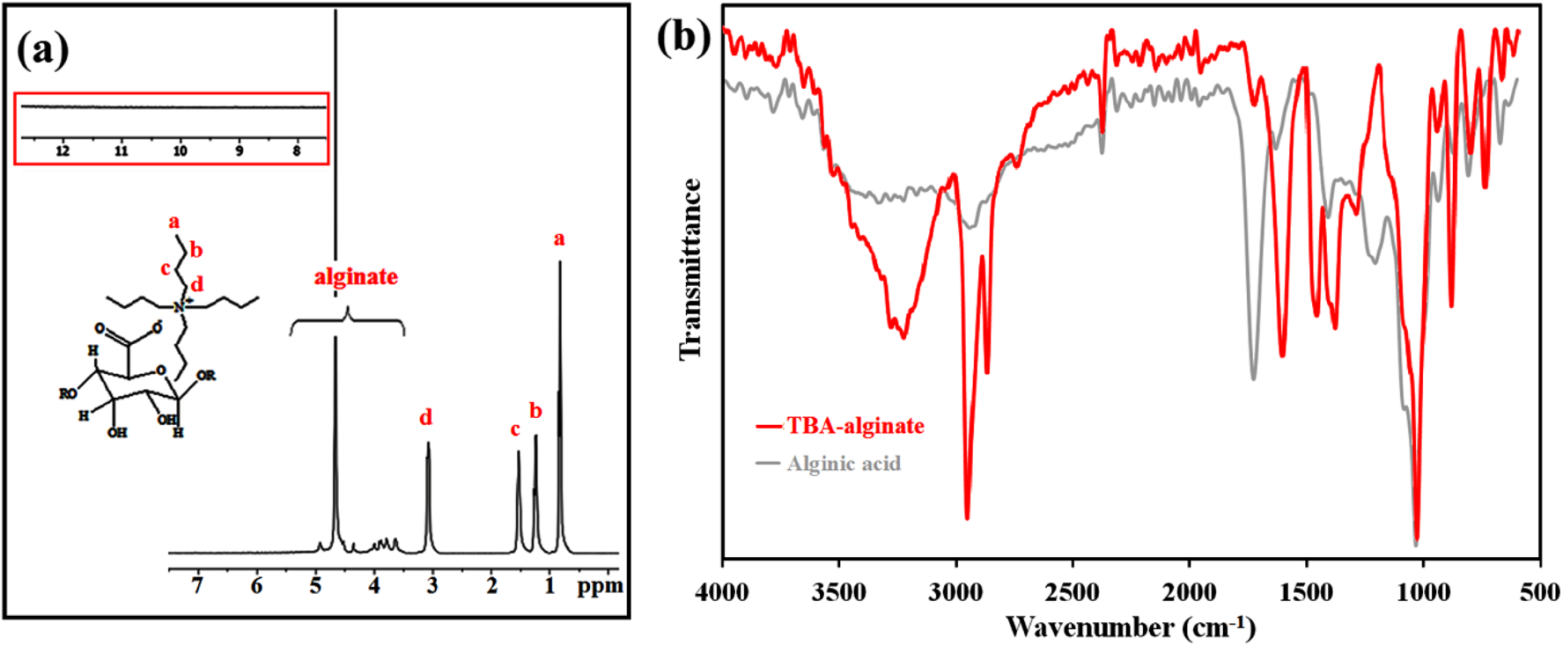
^1^H-NMR (in D_2_O) **(a)** (Inset: TBA-alginate does not exhibit any acidic signal in the downfield region of the ^1^H-NMR spectrum); and FT-IR (**b**) spectra of TBA-alginate (FT-IR spectra of alginic acid is in the background of (**b**)).

In the third step, the LMW-alginic acid was reacted with TBAOH to produce TBA alginate (**TBA**/Alg). ^1^H NMR and FTIR were applied to evaluate the chemical composition of **TBA**/Alg (Fig. 3a and b, respectively**).** From the ^1^H-NMR spectrum shown in Fig. 3a, the structure of TBA/Alg has been confirmed with peaks appearing between 3.4 and 5.00 ppm, which represent alginate backbone hydrogens and four peaks from butyl hydrogens on TBA (0.70 ppm, 3H, t; 1.27 ppm, 2H, m; 1.55 ppm, 2H, m; 3.20 ppm, 2H, m). The FT-IR spectrum of **TBA**/Alg (Fig. 3b) exhibited almost all the FT-IR peaks of alginic acid; plus, two new absorptions at 2956 and 2872 cm^−1^ assigned to the C–H stretching vibrations of the saccharide structure and tetra butyl ammuniom segments. The shifting of carbonyl stretching frequency to the lower wavenumbers (1603 cm^−1^) indicated a successful cation exchange reaction and the reconstitution of carboxylate groups (Fig. 2b).

**Figure 4:**
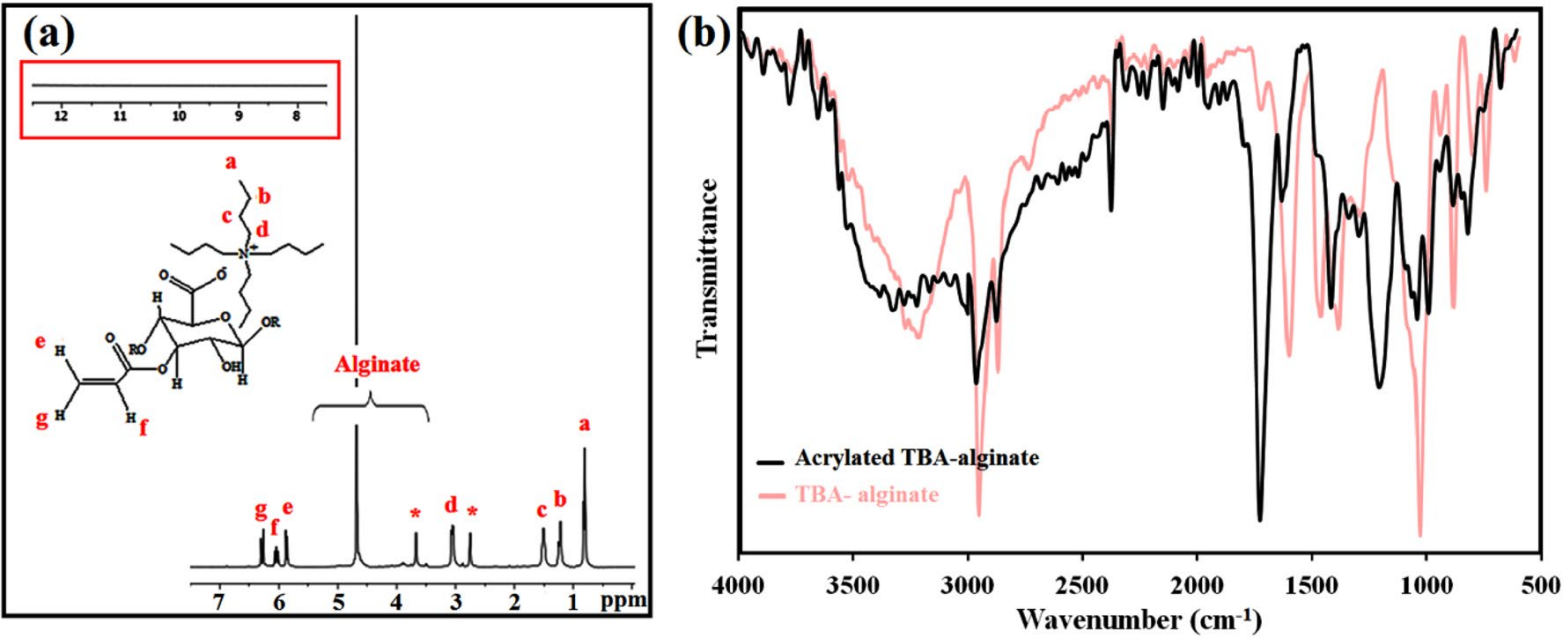
^1^H-NMR (in D_2_O) **(a)** (Inset: Acrylated TBA-alginate does not exhibit any acidic signal in the downfield region of the ^1^H-NMR spectrum), and FT-IR (**b**) spectra of acrylated TBA-alginate (FT-IR spectra of TBA-alginate is in the background of (**b**)).

Functionalization of **TBA**/Alg with acrylate groups was carried out by reacting **TBA**/Alg with acryloyl chloride in the presence of triethylamine under anhydrous and inert conditions, as illustrated in Fig. 2. Successful acrylation is confirmed by the appearance of ^1^H-NMR peaks at δ = 5.8 (i), 6.11 (j), and 6.37 (k) ppm, corresponding to the hydrogens on the acrylate groups (Fig. 4a). This spectrum illustrates all characteristic peaks of **TBA**/Alg between 0.7 and 5.00 ppm. FT-IR analysis was used to elucidate the structure of acrylated TBA alginate. The appearance of two new absorptions at 1724 and 1629 cm^–1^, corresponding to the C=O and C=C stretching bonds of Ac**TBA**/Alg acrylate groups besides the pre-existing peaks of **TBA**/Alg (Fig. 4b) indicated the successful esterification of alginate by acryloyl chloride. The results illustrated that grafting the hydrophobic acryloyl groups on the alginate backbone chain could destroy the original intramolecular hydrogen bonding of alginate and consequently enhance its molecular flexibility^28^.

**Figure 5:**
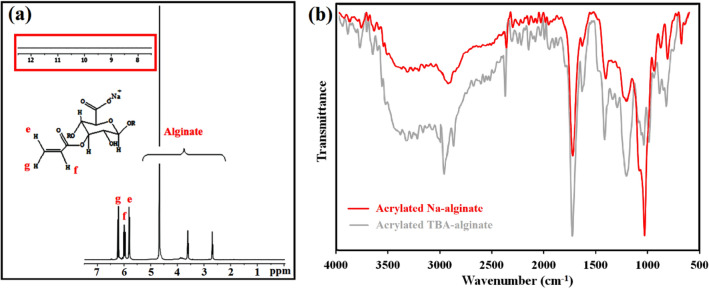
^1^H-NMR (in D_2_O) **(a)** (Inset: Acrylated sodium alginate does not exhibit any acidic signal in the downfield region of the ^1^H-NMR spectrum), and FT-IR (**b)** spectra of acrylated sodium alginate (FT-IR spectra of acrylated TBA-alginate is in the background of (**b**)).

Finally, Ac**TBA**/Alg was reacted with sodium carbonate, and then lyophilized from water. The dry product was characterized by ^1^H-NMR (in D_2_O) and FT-IR spectroscopy. The retained alginate peaks can be seen at 3.7–5.0 ppm, and the TBA fraction was no longer present, as it was removed during neutralization with Na_2_CO_3_ (Fig. 5a). The disappearance of the peak at 1209 cm^–1^ is related to the C4 skeletal stretching of the butyl groups and the acrylate carbonyl group in the FT-IR spectrum of Ac**Na**/Alg spectrum, which indicates the successful neutralization with Na_2_CO_3_ (Fig. 5b).

**Figure 6:**
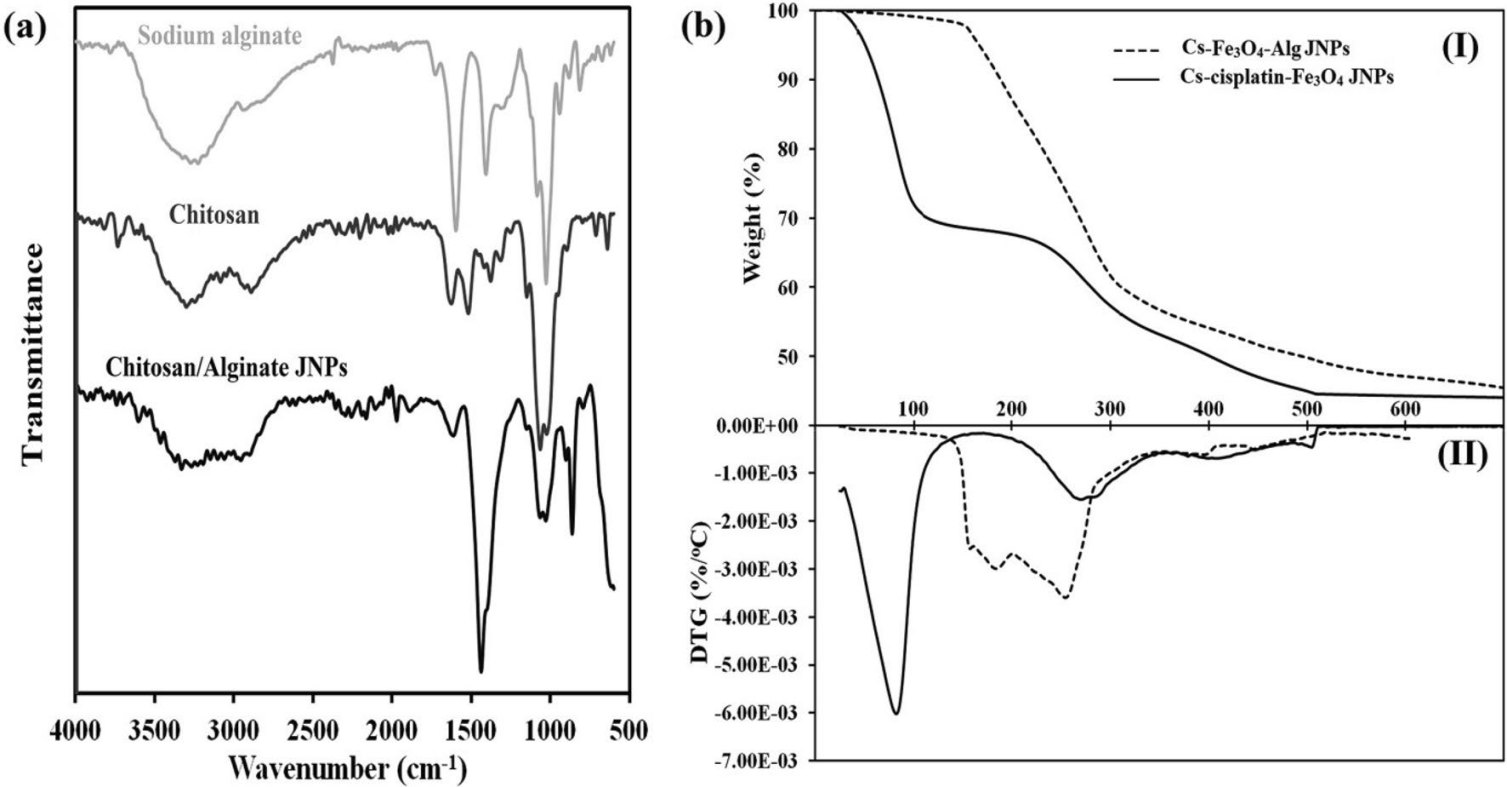
(**a**) FT-IR spectra of sodium alginate, chitosan, and chitosan/sodium alginate Janus nanoparticles, and (**b**) TGA curves of (Cs-cisplatin-Fe_3_O_4_) JNPs and (Cs-Fe_3_O_4_-**Na**/Alg) JNPs at a rate of 10 °C/min.

For the synthesis of chitosan/sodium alginate Janus nanoparticles (Cs-Fe_3_O_4_-**Na**/Alg), at first, Cs-cisplatin-Fe_3_O_4_ JNPs were reacted with APTES and then, the reaction between amine functional groups of Cs-cisplatin-Fe_3_O_4_-APTES JNPs and acrylates functional groups in Ac**Na**/Alg results in the preparation of the Janus nanoparticles (Fig. 1b). FT-IR spectrum of (Cs-Fe_3_O_4_-**Na**/Alg) is shown in Fig. 6a and compared with their pure polymer sources. The strong O–H stretching peak appears at 3400 cm^−1^, which is also observed in pure chitosan spectra. At 2923 cm^−1^ there is a strong peak related to the C–H stretching, which belongs to the alkane groups of the chitosan rings, alginate, and APTES. The ether and ester groups of chitosan and alginate can be distinguished as the peak at 1071 cm^−1^ corresponding to the C–O stretching vibrations. C–N stretching and N–H bending vibrations of amine groups in chitosan are significant as peaks at 1100 and 1600 cm^−1^. The appearance of the absorption bands around 1680, 1416, and 1306 cm^−1^ attributed to stretching vibrations of asymmetric and symmetric bands of Alg carboxylate anions, respectively confirmed the binding of Alg to nanoparticles, and the preparation of Cs-Fe_3_O_4_-**Na**/Alg JNPs.

**Figure 7:**
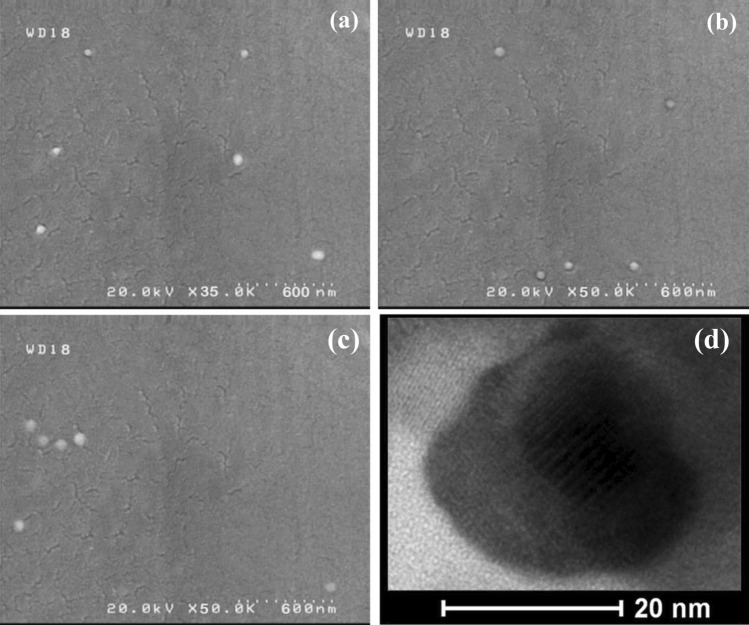
SEM picture of (APTES- Fe_3_O_4_) JNP (**a**), (CS-Fe_3_O_4_) JNP (**b**), (CS- Fe_3_O_4_-**Na**/Alg) JNP (**c**); and TEM image of (Cs-Fe_3_O_4_-**Na**/Alg) JNPs with two distinctly different sides (**d**).

Thermogravimetric analysis (TGA) and derivative thermogravimetric analysis (DTG) were used to determine the amount of chitosan and alginate linked on the surface of NPs. For this purpose, the Janus nanoparticles before alginate adding (i.e. Cs-cisplatin-Fe_3_O_4_ JNPs) and after that (i.e. Cs-Fe_3_O_4_-**Na**/Alg JNPs) were analyzed by TGA (Fig. 6b). In the TGA curve of Cs-cisplatin-Fe_3_O_4_ sample, the first notable weight loss occurred by heating from room temperature to 110 °C which is related to the evaporation of trapped and physisorbed water from the sample (Fig. 6b-I). The second distinct weight loss starts at 217 °C related to the decomposition of conjugated chitosan^29^. The chitosan mass loss is about 23.41%, which means the inorganic fraction to polymeric fraction (chitosan) in the first Janus nanoparticles is 76.59%:23.41%. TGA/DTG thermograms of the second Janus nanoparticles confirms the presence of chitosan and **Na**/Alg domains on the Janus Cs-Fe_3_O_4_-**Na**/Alg surface. The TGA thermogram of Cs-Fe_3_O_4_-**Na**/Alg JNPs exhibited a gradual degradation from 130 to 650 °C (Fig. 6b-I), while, DTG illustrated the first decomposition step in the range of 130–210 °C with a maximum at 184 °C assigned to sodium alginate degradation and a second degradation in the 205–314 °C region with a maximum at 254 °C ascribed to degradation of chitosan and sodium alginate^30,31^ (Fig. 6b-II). According to the TGA curve of Cs-Fe_3_O_4_ -**Na**/Alg, a weight loss of 2.37% was found for residual water. According to the DTG curve, the decomposition of sodium alginate was mixed with the degradation of chitosan before reaching the baseline. The thermal degradation continues gradually to 585 °C. In addition, a total weight loss of 46% was obtained due to the simultaneous degradation of chitosan and sodium alginate. The structure and morphology of the samples were characterized using transmission electron microscopy (TEM) (JEM-1011, JEOL, Japan) and scanning electron microscopy (SEM) (S4800, Hitachi, Japan). The SEM images of all the samples were taken after removing the wax from the surface of these nanoparticles. Figure 7a showed a spherical shape for APTES-Fe_3_O_4_ JNP with an average particle diameter of approximately 48 nm. After coating APTES-Fe_3_O_4_ JNP with Cs and **Na**/Alg, the average particle diameter increased to 56 and 67 nm, respectively, as shown in Fig. 7b and c. HR-TEM image of Cs-Fe_3_O_4_-**Na**/Alg JNPs in Fig. 7d displays the presence of alternated regions constructed from dark and bright areas probably related to **Na**/Alg and Cs polymers, respectively, and supported the formation of Janus structure with an average particle size in the range of 30–60 nm. The size obtained from TEM is in good agreement with that obtained from SEM.

In an attempt to further evaluate the nanoparticle properties, the particle size, polydispersity index (PDI), and surface charge of nanoparticles were determined by DLS. The size distribution of the Fe_3_O_4_ NPs, APTES-Fe_3_O_4_, cisplatin-Fe_3_O_4_, Cs-cisplatin-Fe_3_O_4_, and Cs-Fe_3_O_4_-**Na**/Alg JNPs is shown in Table 1.

**Table 1:** Zeta potential, size and size distribution of the product of different stages of synthesis.

Sample	Zeta potential (mV)^a^	Size (nm)	PDI
Fe_3_O_4_ NP_S_	− 21.6	99.2	0.212
Janus(APTES-Fe_3_O_4_) NP_S_	− 13.9	105.3	0.173
Janus(cisplatin-Fe_3_O_4_) NP_S_	− 13.3	111.3	0.151
Janus(CS-cisplatin-Fe_3_O_4_) NP_S_	4.5	122.6	0.205
Janus(Cs-Fe_3_O_4_-**Na**/Alg) NP_S_	− 30	153.1	0.135

^a^The zeta potential of all nanoparticles was measured at pH 7.4.

The average size of Cs-Fe_3_O_4_-**Na**/Alg JNPs was 132.9 nm with narrow size distribution (PDI = 0.099). Since the polymer chains on the surface of the JNPs swelled in water^32^, the diameter data resulting from the DLS study was much higher than that obtained from SEM and TEM. In the absence of any steric stabilization layer, the naked Fe_3_O_4_ NPs show the zeta potential value of − 21.6 mV at pH 7.4, which showed relatively good stability (Table 1). This value decreased to − 13.9 when half of the surface of Fe_3_O_4_ JNPs was reacted with APTES. The reaction of amine groups of APTES with cisplatin resulted in a new aminated sample; therefore, no significant change was observed in its zeta value. The zeta potential of Cs-cisplatin-Fe_3_O_4_ JNPs is increased at pH = 7.4 due to the high number of amine functional groups present in the chitosan chains, which overcomes the negative charge of iron oxide. The shift of zeta potential n the three last samples was attributed to the presence of amine groups on the surface of the particles^33^. By shielding the other side of the Janus nanoparticle with **Na**/Alg, the zeta potential of Janus nanoparticles shows a reverse trend toward a negative datum and reaches − 30 mV. The decrease in the zeta potential of Alg-coated JNPs is due to the increase in the number of anionic polyelectrolyte COO¯. Thus, Cs-Fe_3_O_4_-**Na**/Alg JNPs with high zeta potential values provide a highly stable system by combining the steric and electrostatic hindrance due to the thick layer of alginate linked to JNPs.

### Self-propelled motion of the nanomotors

**Figure 8:**
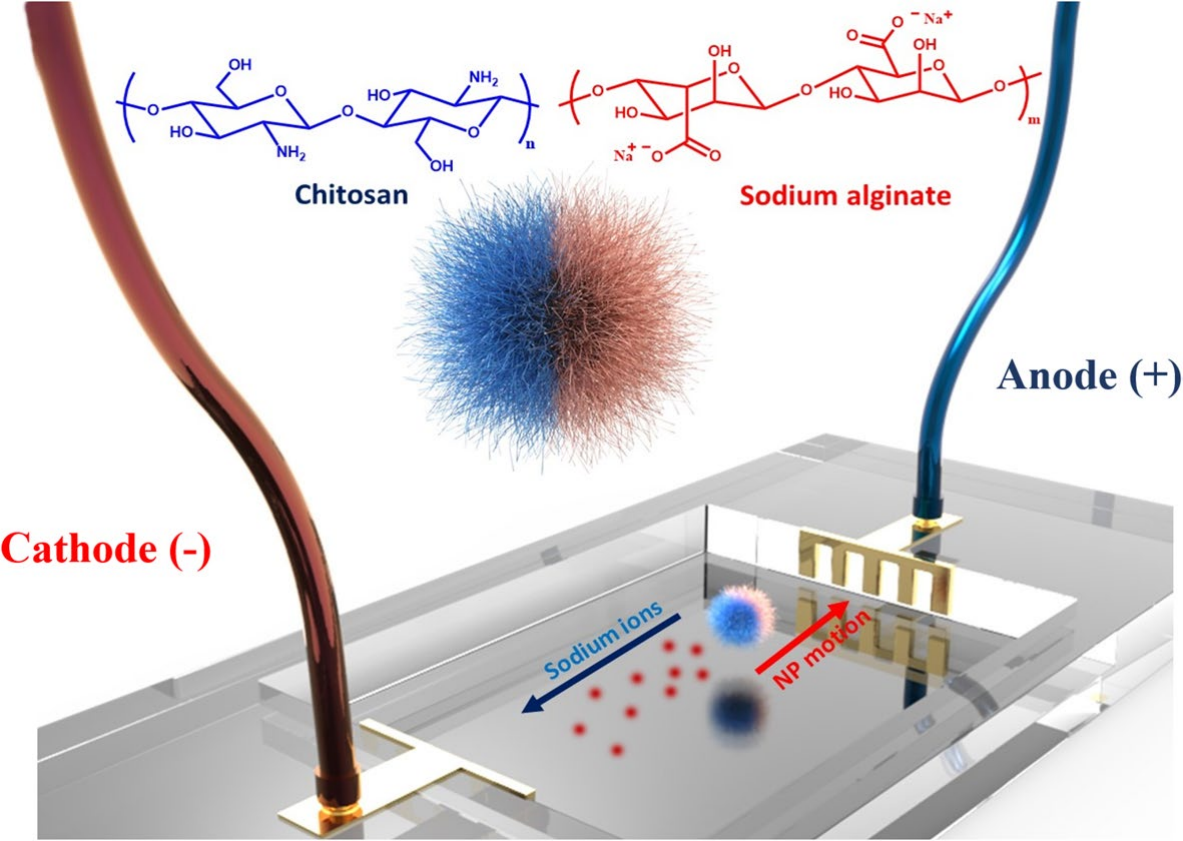
A schematic illustrating the experimental design of coplanar electrodes. The trajectory of the active particles is recorded using video microscopy while applying the DC field (This figure was created by Zbrush: https://pixologic.com/ version: 2021.6.).

To investigate the motion of Janus nano/micro motors, a cell was designed and utilized as shown in Fig. 8. The cell was filled with deionized water and the motors were introduced between two Copper (Cu) electrodes. By applying an electric field through different voltages and pulses, charge separation occurred in the fluidic media containing Janus nanoparticles with at least one polarizable surface. As a result, the nanoparticles began to move in the opposite direction like a swimmer due to induced-charge electroosmosis (ICEO)^34^, where the sodium ions of the alginate domain moved towards the anode electrode while the charged solid particles generated flows around themselves and pushed the surrounding fluid. A digital electronic microscope connected to a digital camera was placed on the top of the chamber for observation purposes.

To investigate the effect of asymmetric ICEO flow around Janus nano/micro motors, we performed systematic experiments in a DC electric field (E) with different voltages and pulses (Videos S1–S5).

As shown in these videos, these JNMs exhibited electrically-driven with precisely steered start/stop switch, speed, and motion trajectory in aqueous media. The trajectories of the particles were analyzed by mean squared displacement (MSD) calculations. For this purpose, once the nanomotors started their motion under the electric field, their movement was recorded with a camera at a speed of 10 frames per second. Tracking the nanomotors' motion was performed with a tracking tool programmed in MATLAB, and the MSD was calculated by Eq. (1):

1 $$MSD\left(\Delta t\right)=\frac{\sum_{i=0}^{n-\Delta t}\left.{\left(({px}_{i}-{px}_{\Delta t+i}\right)}^{2}-{\left(({py}_{i}-{py}_{\Delta t+i}\right)}^{2}\right)}{N+1-\Delta t} ,$$

(N: number of frames; PX and PY are vector positions particles at different times). The diffusion coefficient and velocity were obtained from the plot of MSD vs Δt, through the parabolic component of the Stokes − Einstein equation.

2 $$D=4D\Delta t+{(V\Delta t)}^{2},$$

**Figure 9:**
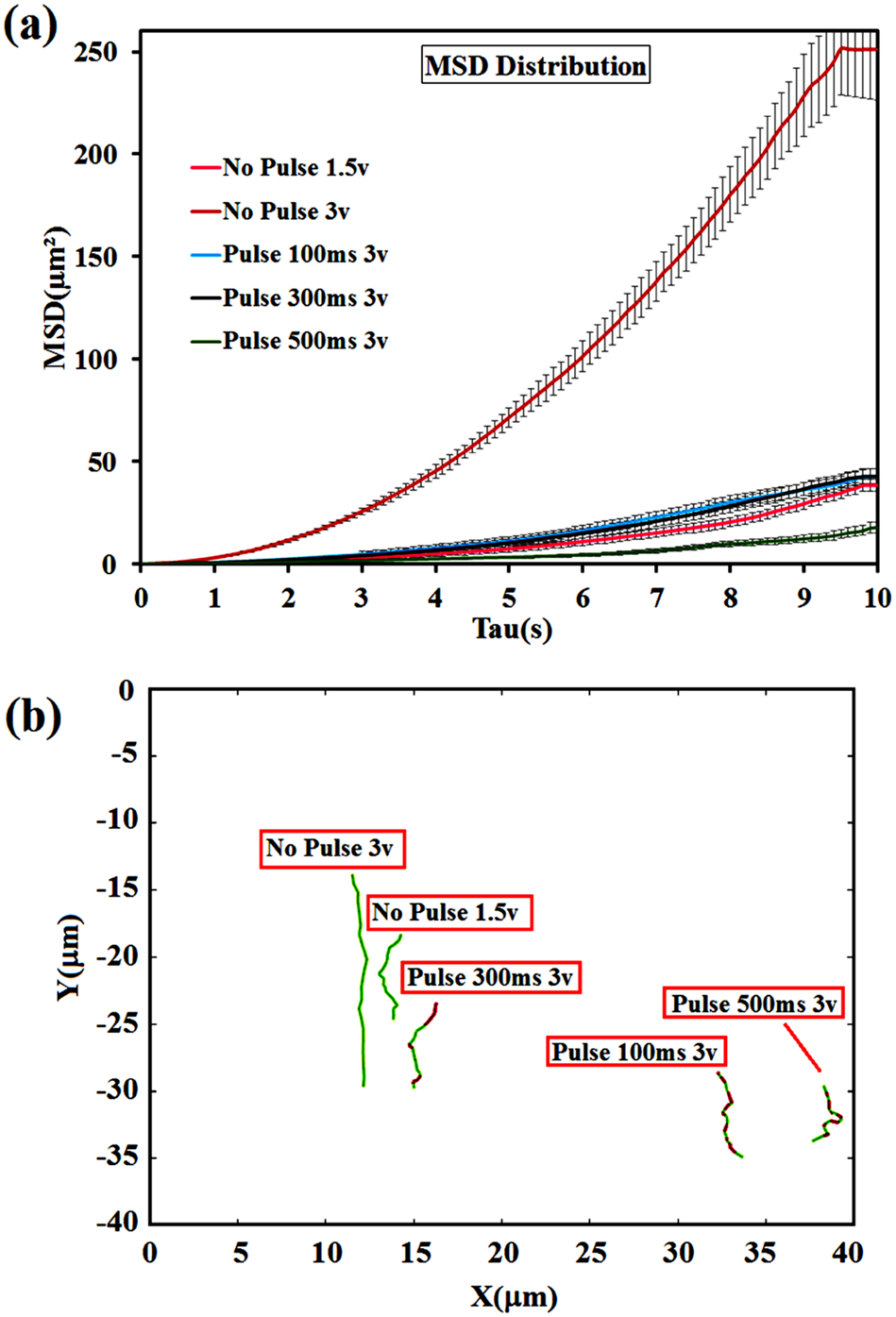
Distributions of MSD values obtained for individual JNPs at the time scale Δt = 10 s under effects of different voltages and pulses (**a**); typical single-particle tracking trajectories of JNMs (**b**), (obtained from Supplementary Videos S1–S5 in the ESI).

Figure 9a and b exhibited the trajectories of JNMs and the related MSD analysis at five different voltages and pulses, respectively. As shown in Table 2**,** the diffusion coefficient and velocity have a direct relation to voltage. So, an increase in their value is observed when the higher voltage is applied because the average (drift) velocity of ions in sodium alginate chains depends on the voltage of the source. Moreover, a significant decrease in the diffusion coefficient and velocity was observed by applying the pulse, and its MSD diagram became almost similar to the Brownian motion MSD diagram^35^.

**Table 2:** Diffusion coefficient (D) values obtained for individual JNPs at the time scale Δt = 10 s (obtained from Supplementary Videos S1–S5 in the ESI).

Voltage	D (µm^2^/s)	V (µm/s)	Video #
No pulse 1.5v	0.64 + 0.0132	0.64 + 0.0068	S1
No pulse 3v	5.20 + 0.0365	1.64 + 0.0143	S2
Pulse 100 ms 3v	0.85 + 0.0164	0.74 + 0.0083	S3
Pulse 300 ms 3v	0.82 + 0.0081	0.79 + 0.0052	S4
Pulse 500 ms 3v	0.28 + 0.0065	0.68 + 0.0095	S5

In the next step, we investigated the effect of di- and trivalent metal cations (Fe^3+^, Al^3+^, Ba^2+^, Ca^2+^ and Mg^2+^) as alginate cross-linkers. The effects of this parameter were analyzed by considering the nanoparticle size and the nanomotors’ speed. The nanoparticles crosslinked with divalent cations (Ba^2+^, Ca^2+^, Mg^2+^) and trivalent cations (Al^3+^ and Fe^3+^) were prepared by dispersing Cs-Fe_3_O_4_-**Na**/Alg JNPs in the aqueous solutions containing either BaCl_2_, CaCl_2_, MgCl_2_, FeCl_3_ or Al_2_(SO_4_)_3_ salts. The effect of Fe^3+^, Al^3+^, Ba^2+^, Ca^2+^, and Mg^2+^ ions on sodium alginate domains was analyzed by determining the radius of nanoparticles through DLS method. According to the results obtained from the intensity mode of DLS, of aqueous suspensions of Janus nanoparticles, we can conclude that the process of binding of metal ions occurs via crosslinking of alginate by di and trivalent metal cations (Table 3).

**Table 3:** Size and size distribution of Janus nanoparticles cross-linked by various ions.

Cations		Ions Pauling radius (pm)^a^	Janus nanoparticles	Size (nm) ^b^	PDI ^b^
Divalent ions	Magnesium (Mg^2+^)	65	(CS-Fe_3_O_4_-**Mg**/Alg)	138.9	0.640
	Calcium (Ca^2+^)	99	(CS-Fe_3_O_4_-**Ca**/Alg)	145.6	0.768
	Barium (Ba^2+^)	135	(CS-Fe_3_O_4_-**Ba**/Alg)	159.3	0.205
Trivalent ions	Aluminium (Al^3+^)	50	(CS-Fe_3_O_4_-**Al**/Alg)	118.7	0.085
	Ferric (Fe^3+^)	64	(CS-Fe_3_O_4_-**Fe**/Alg)	112.3	0.270
Monovalent ions	Lithium (Li^+^)	60	(Cs-Fe_3_O_4_-**Li**/Alg)	149.4	0.153
	Sodium (Na^+^)	95	(Cs-Fe_3_O_4_-**Na**/Alg)	156.1	0.327
	Potassium (K^+^)	133	Cs-Fe_3_O_4_-**K**/Alg)	166.1	0. 859

^a^This column shows ions Pauling radii for Li^+^, Na^+^, K^+^, Mg^2+^, Ca^2+^, Ba^2+^, Al^3+^, and Fe^3+^, respectively^36^. ^b^ The results were obtained by dynamic light scattering (DLS) measurements.

By examining the size in terms of time, it is clear that the alginate part of Janus nanoparticles rapidly produces gel by creating intermolecular interactions with divalent and trivalent metal cations (BaCl_2_, CaCl_2_, MgCl_2_, FeCl_3_ or Al_2_(SO_4_)_3_). The results show that the effect of trivalent ions on size reduction is greater than divalent ions and that of divalent ions are more efficient than monovalent ones.

We expected that the size of nanoparticles will affect the speed of the nanomotor cross-linked with each of these ions. For this purpose, the movements of nanomotors pre-suspended in different ions with equimolar concentration were recorded by the camera (Videos S6–S12), and their velocities were evaluated by MSD. As demonstrated in Table 4, adding divalent and trivalent ions with equal molar concentration to the aqueous solutions of nanomotors based on sodium alginate increases the speed of the nanomotors under the electrical field. This increase is higher for trivalent ions related to divalent ones because the trivalent ions provide more charge in the cell media and, as a result, create a higher driving force for moving the nanoparticles^37^. But what surprised us was the increase in speed by increasing the atomic weight in each group. Harlaftis et al. demonstrated that for spherical nanoparticles, the dielectrophoretic forces depend linearly on the particle volume, when external electric field, field density gradient, and frequency are constant^38^. So, according to this theory, in samples with identical ion charges, the increase in the speed according to the particle radius is logical.

**Table 4:** Diffusion coefficient (D) and velocity (V) values obtained for individual JNPs with different cations at the time scale Δt = 9 s, under effects of voltage (1.5v) without pulse (obtained from Supplementary Videos S6–S12 in the ESI).

Cations	Janus nanoparticles	D (µm^2^/s)	V (µm/s)	Video #
Divalent ions	(CS-Fe_3_O_4_-**Mg**/Alg)	0.94481 ± 0.0048	1.1081 ± 0.0012	S6
	(CS-Fe_3_O_4_-**Ca**/Alg)	1.9591 ± 0.025	1.5005 ± 0.0048	S7
	(CS-Fe_3_O_4_-**Ba**/Alg)	4.2267 ± 0.0059	2.061 ± 0.0073	S8
Trivalent ions	(CS-Fe_3_O_4_-**Al**/Alg)	2.5747 ± 0.0038	1.7459 ± 0.069	S9
	(CS-Fe_3_O_4_-**Fe**/Alg)	7.2181 ± 0.0084	2.7767 ± 0.0025	S10
Monovalent ions	(Cs-Fe_3_O_4_-**Li/**Alg)	1.6221 ± 0.0058	2.2282 ± 0.0076	S11
	(Cs-Fe_3_O_4_-**Na**/Alg)	0.64 ± 0.0132	0.64 ± 0.0068	S1
	(Cs-Fe_3_O_4_-**K**/Alg)	3.5962 ± 0.0017	2.7282 ± 0.0083	S12

**Figure 10:**
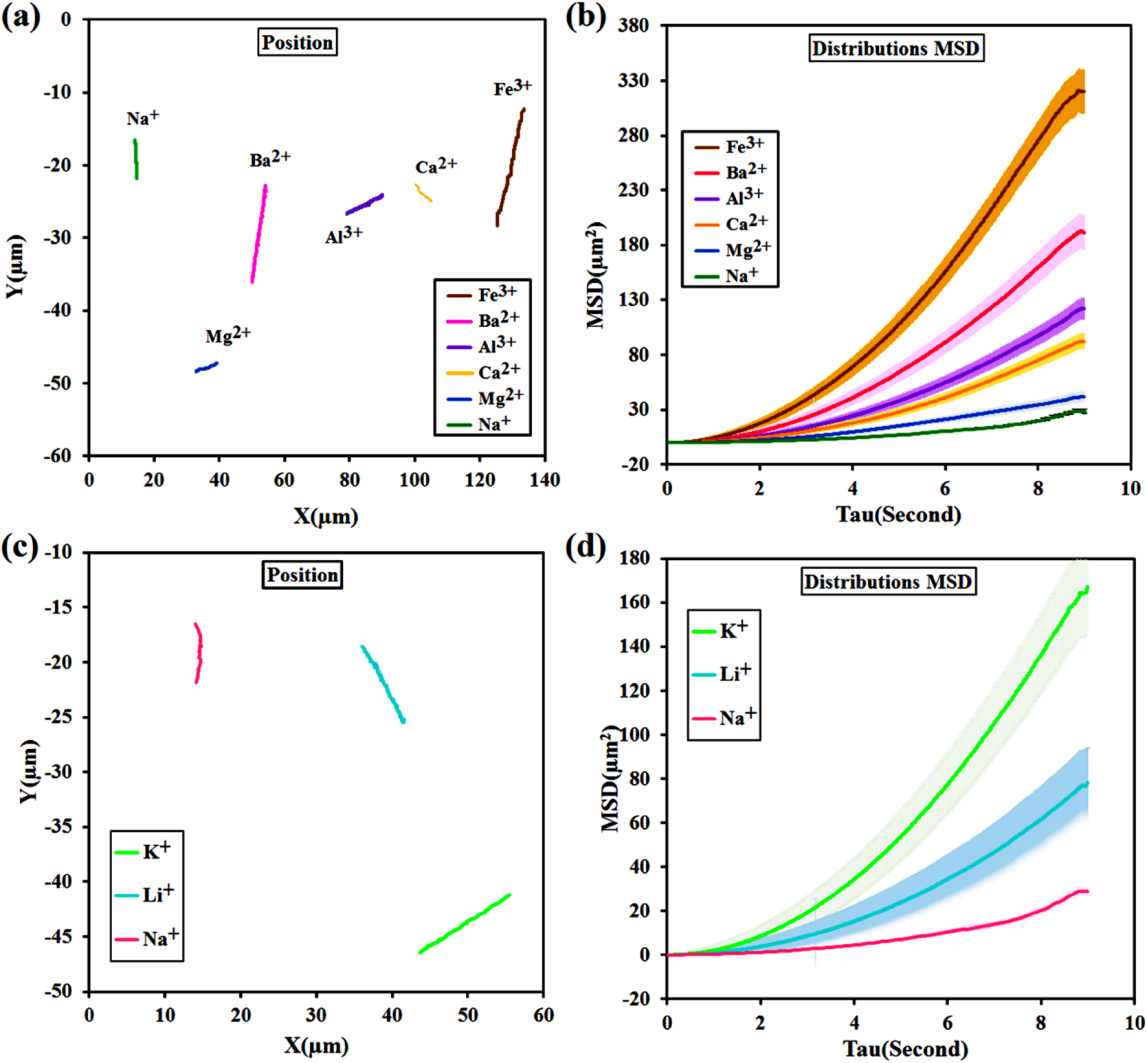
Typical single-particle tracking trajectories of JNMs in the presence of different multi valent and monovalent cations as crosslinker agents for n = 30 trajectories ((**a**) di- and ti-valent cations; (**c**) mono-valent cations, obtained from Videos S6–S12), and the distributions of MSD values of the corresponding JNPs ((**b**) di- and ti-valent cations; (**d**) mono-valent cations) at the time scale Δt = 9 s under effects of voltage (1.5v) without pulse.

Figure 10a shows the trajectory of JNMs with different cationic agents as binders in an observation time of about 9 s. As can be seen, when Na^+^ was replaced by Fe^3+^, the path traveled by JNMs increased from 6.4 to 24.99 μm. MSD analysis shows a semi-parabolic trend of nonlinear relationship, and its tangent slope gradually increases. Results obtained in the study showed that velocities of motion are 2.7767 ± 0.0025, 1.7459 ± 0.069, 2.061 ± 0.0073, 1.5005 ± 0.0048, 1.1081 ± 0.0012 µm/s for Fe^3+^, Al^3+^, Ba^2+^, Ca^2+^, and Mg^2+^ respectively (Fig. 10b).

In the next step, the speed of nanomotors with some other monovalent ions was analyzed to evaluate the Harlaftis theory. For this purpose, potassium or lithium salts were added to the solution containing sodium alginate-based JNMs, in the same amount as di/trivalent ions and their movements were recorded (Videos S11 and S12). KCl and LiCl salts are not cross-linking agents, so the largest particle size is seen for nanomotors with these ions (Table 3). Interestingly, after Fe^3+^, the highest speed is for nanomotors containing Li and K (Table 4, Fig. 10c and d). In conclusion, it can be said that the amount of charge and particle size are both effective parameters, but more theoretical/experimental studies are needed to investigate the effect rate of each of these two parameters on the movement speed of nanomotors. Upon examination of the time interval investigated in this study, it can be observed that all samples exhibit enhanced diffusion behavior which follows a ballistic motion. The mean squared displacement (MSD) of the particles exhibits a non-linear increase with time, as evidenced by the relationship ⟨Δx^2^⟩ ∼ Δt^2^. This phenomenon is indicative of superdiffusive transport, which occurs due to the propulsion of the particles under the influence of an electric field^39^.

The only item that revealed a high inconsistency with other data in Table 4 is related to nanomotors containing sodium ions. Because, the only cation present in sodium alginate-based nanomotors is sodium, while in nanomotors with other cations, in addition to the arrival ions (ions replaced instead of sodium), some departure ions (sodium ions) also exist. Therefore, the presence of extra salts beside the sodium ions increased the ionic concentration of the solution. As a result, the motor moving speed becomes higher when the ionic concentration rises due to the increase in the generated driving forces^37^. JNMs with electrical propulsion mechanisms combined with a magnetic field as an external source are prevalent, powerful, and highly efficient. Incorporating magnetic elements iron (III) oxide into JNMs allows for accurate motion control of nanomotors. We next evaluated the micromotor propulsion in magnetic mode. The locomotive behavior of Janus(Cs-Fe_3_O_4_-Na/Alg) nanomotors by an external magnetic field was recoded via camera (Video S13). A change in the trajectory with directional movement can be observed by applying the magnetic field (Fe_3_O_4_ engine). The magnetic force changes the flow rate around the Janus micromotors, thereby increasing the acceleration of the micromotor, which could be very useful for future applications in complex media. It should be emphasized that the remarkably high velocity exhibited by these nanomotors posed a significant challenge in terms of accurately tracking their movements and conducting meaningful MSD analysis.

## Conclusions

Here, we report an electrically powered Janus micromotor based on SPION that moves in aqueous media. This Janus nanomotor consists of a uniform spherical SPION as the core and two hemispheres of **Na**/Alg and Cs as the shell. Based on the electroactive properties of sodium alginate, the micro/nanomotor not only moves in solution under the influence of an electric field based on the electroosmotic mechanism, but also shows the great versatility in regulating the movement behavior under the influences of the electrical field. The electrical field controls the motion direction and speed of Janus micro/nanomotor in aqueous media on-demand. In addition, we found that the movement speed of nanoparticles increases with increasing the voltage between the two electrodes. The addition of divalent and trivalent ions as crosslinking agents affects both the size of nanomotors and the total charge of the medium. Increasing the amount of either of these two parameters could increase the final speed of the nanomotors. Therefore, the final velocity observed for each nanoparticle is the cross-product of the vectors of these two parameters. The highest speed of JNMs is observed when Fe^3+^ is used as crosslinking agent (about 7.2181 μm^2^ /s). As well as, the addition of non-crosslinking LiCl and KCl salts to the aqueous media causes an enhancement in the propulsion of microswimmers because in the presence of these monovalent ions, the size of nanoparticles -as an influencing parameter on velocity- is much higher than di/trivalent ions.

## Supplementary Information

Supplementary Information.

Supplementary Video 1.

Supplementary Video 2.

Supplementary Video 3.

Supplementary Video 4.

Supplementary Video 5.

Supplementary Video 6.

Supplementary Video 7.

Supplementary Video 8.

Supplementary Video 9.

Supplementary Video 10.

Supplementary Video 11.

Supplementary Video 12.

Supplementary Video 13.

## Data Availability

The authors confirm that the data supporting the findings of this study are available within the article.

## References

[1] Gao W (2015). Artificial micromotors in the mouse’s stomach: A step toward in vivo use of synthetic motors. ACS Nano.

[2] Li J (2014). Water-driven micromotors for rapid photocatalytic degradation of biological and chemical warfare agents. ACS Nano.

[3] Demirok UK, Laocharoensuk R, Manesh KM, Wang J (2008). Ultrafast catalytic alloy nanomotors. Angew. Chem. Int. Ed..

[4] Gao W, Pei A, Dong R, Wang J (2014). Catalytic Iridium-based Janus micromotors powered by ultralow levels of chemical fuels. J. Am. Chem. Soc..

[5] Ma X, Wang X, Hahn K, Sanchez S (2016). Motion control of urea-powered biocompatible hollow microcapsules. ACS Nano.

[6] Jang B (2017). Multiwavelength light responsive Au/BTiO2 Janus micromotors. ACS Nano.

[7] Zhang L (2009). Characterizing the swimming properties of artificial bacterial flagella. Nano Lett..

[8] Ma F, Yang X, Zhao H, Wu N (2015). Inducing propulsion of colloidal dimers by breaking the symmetry in electrohydrodynamic flow. Phys. Rev. Lett..

[9] Bregulla AP, Yang H, Cichos F (2014). Stochastic localization of microswimmers by photon nudging. ACS Nano.

[10] Wang W, Castro L, Hoyos AM, Mallouk TE (2012). Autonomous motion of metallic microrods propelled by ultrasound. ACS Nano.

[11] Pal M (2018). Maneuverability of magnetic nanomotors inside living cells. Adv. Mater..

[12] Xu T (2015). Reversible swarming and separation of self-propelled chemically powered nanomotors under acoustic fields. J. Am. Chem. Soc..

[13] Li J (2015). Magneto–acoustic hybrid nanomotor. Nano Lett..

[14] Bayati P, Najafi A (2019). Electrophoresis of active Janus particles. J. Chem. Phys..

[15] Zhuang R (2022). Alternating current electric field driven topologically defective micro/nanomotors. Appl. Mater. Today.

[16] Liang Z, Teal D, Fan D (2019). Light programmable micro/nanomotors with optically tunable in-phase electric polarization. Nat. Commun..

[17] Wu Y, Yakov S, Fu A, Yossifon G (2023). A magnetically and electrically powered hybrid micromotor in conductive solutions: Synergistic propulsion effects and label-free cargo transport and sensing. Adv. Sci..

[18] Xiao Z, Duan S, Xu P, Cui J, Zhang H, Wang W (2020). Synergistic speed enhancement of an electric-photochemical hybrid micromotor by tilt rectification. ACS Nano.

[19] Liang Z, Tu Y, Peng F (2021). Polymeric micro/nanomotors and their biomedical applications. Adv. Healthc. Mater..

[20] Wang Z, Wang Z, Li J, Tian C, Wang Y (2020). Active colloidal molecules assembled via selective and directional bonds. Nat. Commun..

[21] Lee JG, Brooks AM, Shelton WA, Bishop KJM, Bharti B (2019). Directed propulsion of spherical particles along three dimensional helical trajectories. Nat. Commun..

[22] Yang Q (2020). Recent advances in motion control of micro/nanomotors. Adv. Intell. Syst..

[23] Lee JG, Al Harraq A, Bishop KJ, Bharti B (2021). Fabrication and electric field-driven active propulsion of patchy microellipsoids. J. Phys. Chem. B.

[24] Homaeigohar S, Tsai TY, Young TH, Yang HJ, Ji YR (2019). An electroactive alginate hydrogel nanocomposite reinforced by functionalized graphite nanofilaments for neural tissue engineering. Carbohydr. Polym..

[25] Kapishon V, Whitney RA, Champagne P, Cunningham MF, Neufeld RJ (2015). Polymerization induced self-assembly of alginate based amphiphilic graft copolymers synthesized by single electron transfer living radical polymerization. Biomacromolecules.

[26] Mafakheri F, Khoee S (2022). Synthesis of Candida Antarctica Lipase B (CALB) enzyme-powered magnetite nanomotor based on PCL/Chitosan Janus nanostructure. Sci. Rep..

[27] González-López MA (2020). Reducing the effective dose of cisplatin using gold nanoparticles as carriers. Cancer Nano.

[28] Feng M (2018). Synthesis of a benzylgrafted alginate derivative and its effect on the colloidal stability of nanosized titanium dioxide aqueous suspensions for Pickering emulsions. RSC Adv..

[29] Ates B (2018). Magnetic-propelled Fe_3_O_4_-chitosan carriers enhance l-asparaginase catalytic activity: A promising strategy for enzyme immobilization. RSC Adv..

[30] Kulig D, Zimoch-Korzycka A, Jarmoluk A, Marycz K (2016). Study on alginate–chitosan complex formed with different polymers ratio. Polymers.

[31] Işıklan N, Kurşun F, İnal M (2010). Graft copolymerization of itaconic acid onto sodium alginate using benzoyl peroxide. Carbohydr. Polym..

[32] Daryasari MP, Akhgar MR, Mamashli F, Bigdeli B, Khoobi M (2016). Chitosan-folate coated mesoporous silica nanoparticles as a smart and pH-sensitive system for curcumin delivery. RSC Adv..

[33] Bondarenko LS (2020). Effects of modified magnetite nanoparticles on bacterial cells and enzyme reactions. Nanomaterials.

[34] Squires TM, Bazant MZ (2004). Induced-charge electro-osmosis. J. Fluid Mech..

[35] Díez P (2021). Ultrafast directional Janus Pt–mesoporous silica nanomotors for smart drug delivery. ACS Nano.

[36] Marcus Y (1983). Ionic radii in aqueous solutions. J. Solut. Chem..

[37] Dalal M, Dalal M (2018). Electrochemistry–II: Ion transport in solutions. A Textbook of Physical Chemistry.

[38] Harlaftis F (2022). Trapping plasmonic nanoparticles with MHz electric fields. Appl. Phys. Lett..

[39] Orozco J (2014). Bubble-propelled micromotors for enhanced transport of passive tracers. Langmuir.

