# Supplementary material for: Synthesis of magnetic electroactive nanomotors based on sodium alginate/chitosan and investigation the influence of the external electric field on the mechanism of locomotion
Source: Sci Rep. 2023 Jun 26;13:10326. doi: 10.1038/s41598-023-37463-9 (PMC10293247; doi:10.1038/s41598-023-37463-9)
Supplement: Supplementary file 1 — Supplementary Information. [file 41598_2023_37463_MOESM1_ESM.docx]

**Supporting Information**

**Synthesis of magnetic electroactive nanomotors based on sodium alginate/chitosan and investigation the influence of the external electric field on the mechanism of locomotion**

**Fariba Mafakheri, Ali Asakereh, Sepideh Khoee*, and Mojtaba Kamankesh**

Polymer Laboratory, School of Chemistry, College of Science, University of Tehran,

PO Box 14155-6455, Tehran, Iran

* Email: [khoee@ut.ac.ir](mailto:khoee@ut.ac.ir)

**Table of Contents**

# S1. Experimental Procedures

S1.1. Synthesis of Fe_3_O_4_ nanoparticles

S1.2. Preparation of Fe_3_O_4_-wax composite microsphere

S1.3. Modifying waxed SPIONs with APTES

S1.4. Synthesis of cisplatin-Fe_3_O_4_-wax microparticles

S1.5. Synthesis of Cs-cisplatin-Fe_3_O_4_-wax microparticles

S1.6. Removing the wax from the Cs-cisplatin-Fe_3_O_4_-wax microparticles

# S2. Additional Data

# S1. Experimental Procedures

**S1.1. Synthesis of Fe_3_O_4_ nanoparticles**

Magnetite nanoparticles were synthesized via co-precipitation of Fe (II) and Fe (III) chloride in alkali solution according to the previously reported technique [1]. Brieﬂy, 1g of FeCl_2_.4H_2_O and 2.6 g of FeCl_3_.6H_2_O were dissolved in 25 mL distilled water under N_2_ purging and kept at 75 ^0^C for 10 min, then 10 mL of ammonia solution (25%) was added into the prepared mixture dropwise under vigorous stirring and kept at 75 °C for 1.5 h. The resulting mixture was washed three times with distilled water and ethanol and dried under vacuum.

**S1.2. Preparation of Fe_3_O_4_-wax composite microsphere**

Following the theory of Pickering emulsions, a homogeneous emulsion of Fe_3_O_4_/wax was first obtained by addition of paraffin wax (5g) to the mixture of well-dispersed SPIONs (300 mg) in ultrapure water (30 mL) under severe mechanical stirring at 78 °C for 1.5 h [2]. Then the homogeneous emulsions were cooled with pouring the mixture in cold water and was washed with water flow and then ethyl alcohol several times to remove the weakly attached SPION and free wax balls, respectively. Finally, the desired Fe_3_O_4_/wax composite microspheres were kept in ethyl alcohol in cold condition for the next steps.

**S1.3. Modifying waxed SPIONs with APTES (APTES- Fe_3_O_4_-wax microparticles)**

The freshly prepared wax spheres (5g) were dispersed in 100 mL of an ethanol solution containing APTES (2.22 mmol, 0.5 mL) and H­_2_O (0.5 mL), then the stirring was continued at room temperature. Silanization of the exposed surface of the SPIONs was done in 8 h. after the completion of the reaction, the mixture was washed with pure ethanol to remove excess amount of APTES, and the Fe_3_O_4_-wax microspheres that are modified with amino groups (APTES- Fe_3_O_4_-wax) were kept in ethanol for further reactions.

### **S1.4. Synthesis of cisplatin-Fe_3_O_4_-wax microparticles**

Cisplatin (30 mg, 0.08 mmol) was dissolved in 30 mL water and added to a flask containing 3g of APTES- Fe_3_O_4_-wax microparticles dispersed in 30 mL of water. NaHCO_3_ (13 mg, 0.15 mmol) was added to the flask and let to stir for 72 h. As the freshly prepared wax balls are floated in water and sedimented in ethanol, therefore purification was done in water and preservation was performed in ethanol. The produced cisplatin-Fe_3_O_4_-wax microparticles were washed with distilled water using a separating funnel to remove unreacted cisplatin and finally were collected in ethanol using an external magnetic field. Collected cisplatin- Fe_3_O_4_-wax microspheres were stored in ethyl alcohol.

**S1.5. Synthesis of Cs-cisplatin-Fe_3_O_4_-wax microparticles**

Chitosan (20 mg) was dissolved in 7 mL of water and added to a dispersion of cisplatin- Fe_3_O_4_-wax (3g) in ethanol (10 mL) afterward stirred under a stirring rate of 700 rpm at room temperature for 72 h. Prepared Cs-cisplatin-Fe_3_O_4_-wax microspheres were then collected using an external magnetic field and washed with pure ethanol and distilled water to remove the excess amount of chitosan. The product was kept in ethyl alcohol.

### **S1.6. Removing the wax from the Cs-cisplatin-Fe_3_O_4_-wax microparticles**

The Cs-cisplatin-Fe_3_O_4_ nanoparticles were brought out of the wax microsphere by dissolving the nanoparticle-containing wax balls in an excess amount of chloroform through the “bain marie” technique at 100 ^0^C. Chloroform solves the wax and separates the nanoparticles from the wax microsphere. Wax-free nanoparticles were collected using an external magnetic field. Finally, the half-coated Cs-cisplatin-Fe_3_O_4_ nanoparticles were washed several times with chloroform, and then they were kept in ethyl alcohol for use in the next step.

**References**

1. S. Khoee, Y. Bagheri and A. Hashemi, Composition controlled synthesis of PCL–PEG Janus nanoparticles: magnetite nanoparticles prepared from one-pot photo-click reaction, Nanoscale 7 (2015) 4134-4148. <https://doi.org/10.1039/C4NR06590E>.

[2] S. Khoee, A. Keivanshokouh, Anisotropic modification of SPIONs surface with thiol and alkyne groups for fabrication of poly (2-hydroxyethyl methacrylate)/polydopamine amphiphilic Janus nanoparticles via double-click reaction, Colloids Surf. A Physicochem. Eng. 597 (2020) Article number 124777. <https://doi.org/10.1016/j.colsurfa.2020.124777>.

# S2. Additional Data


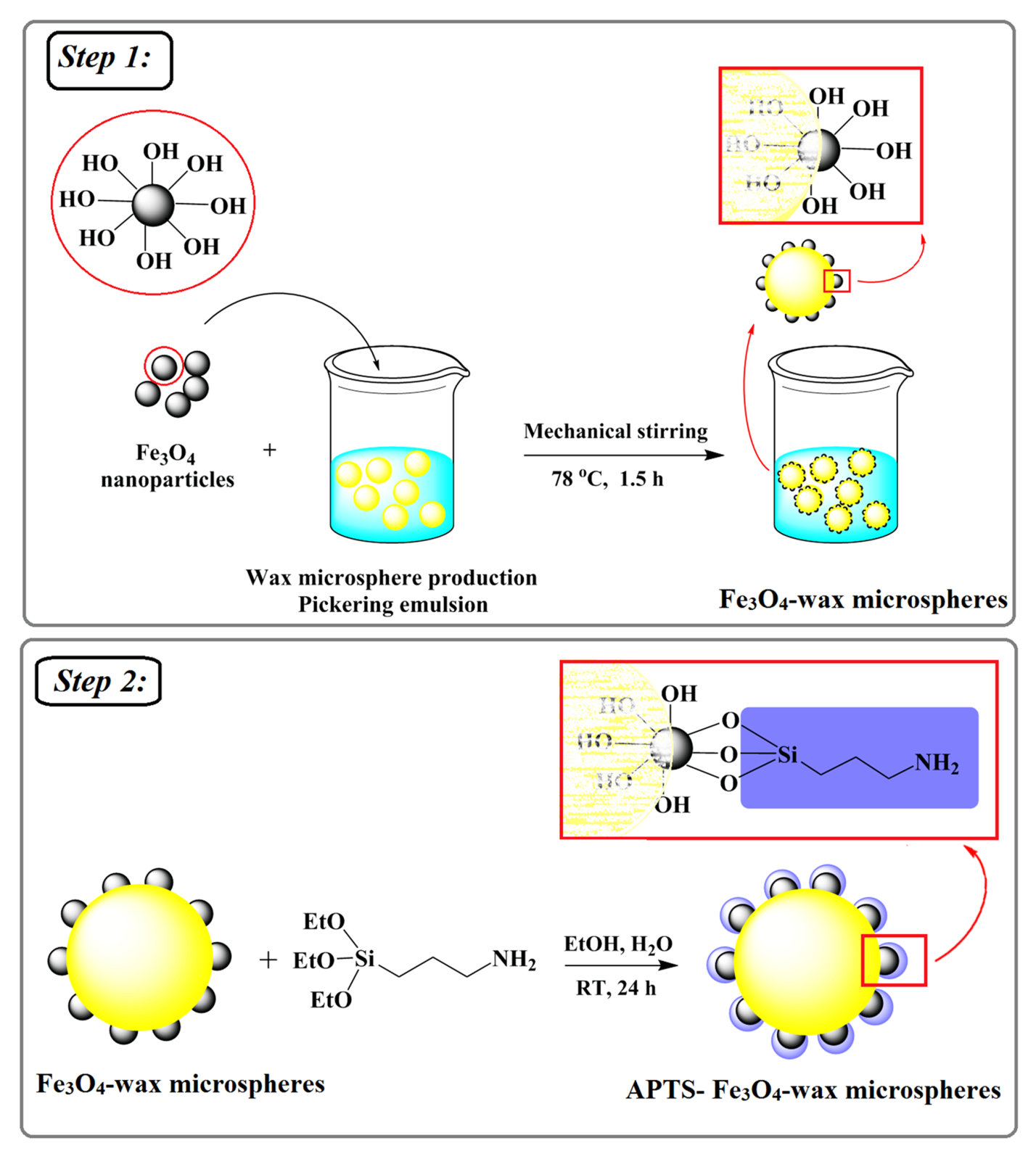


**Fig. S1.** Summary of synthetic route for preparation of: (a) Fe_3_O_4_-wax micrparticles and (b) APTES-Fe_3_O_4_-wax micrparticles


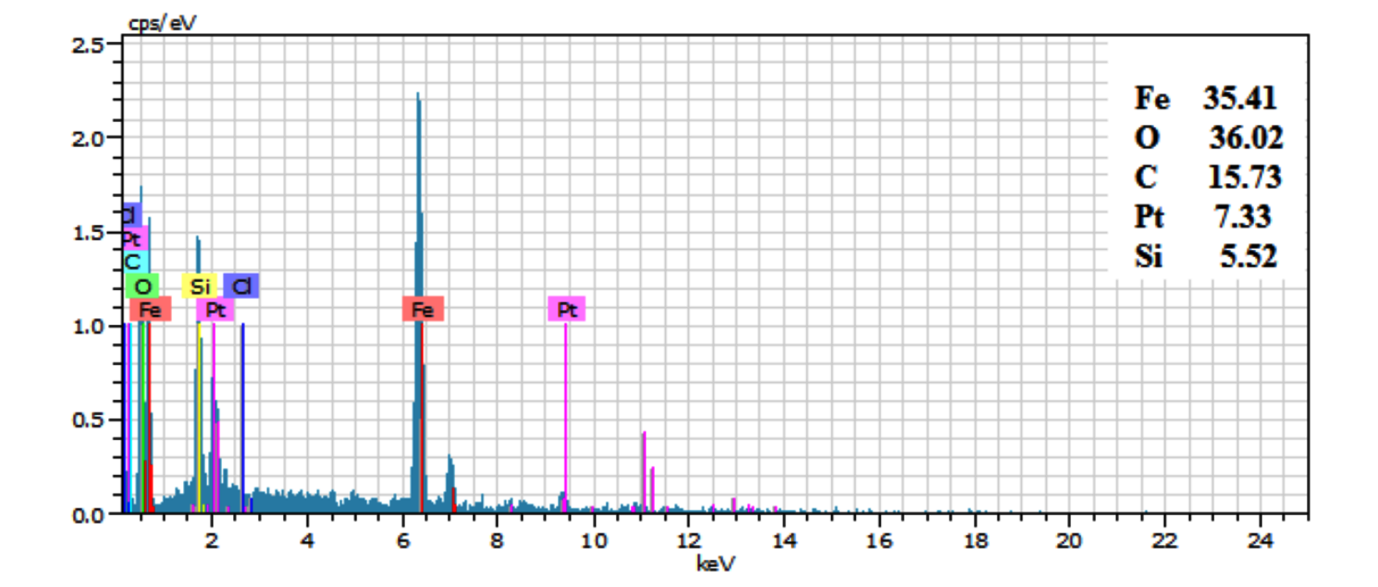


**Fig. S2** Energy-dispersive X-ray (EDX) spectrum of wax-free cisplatin-Fe_3_O_4_ Janus nanoparticle


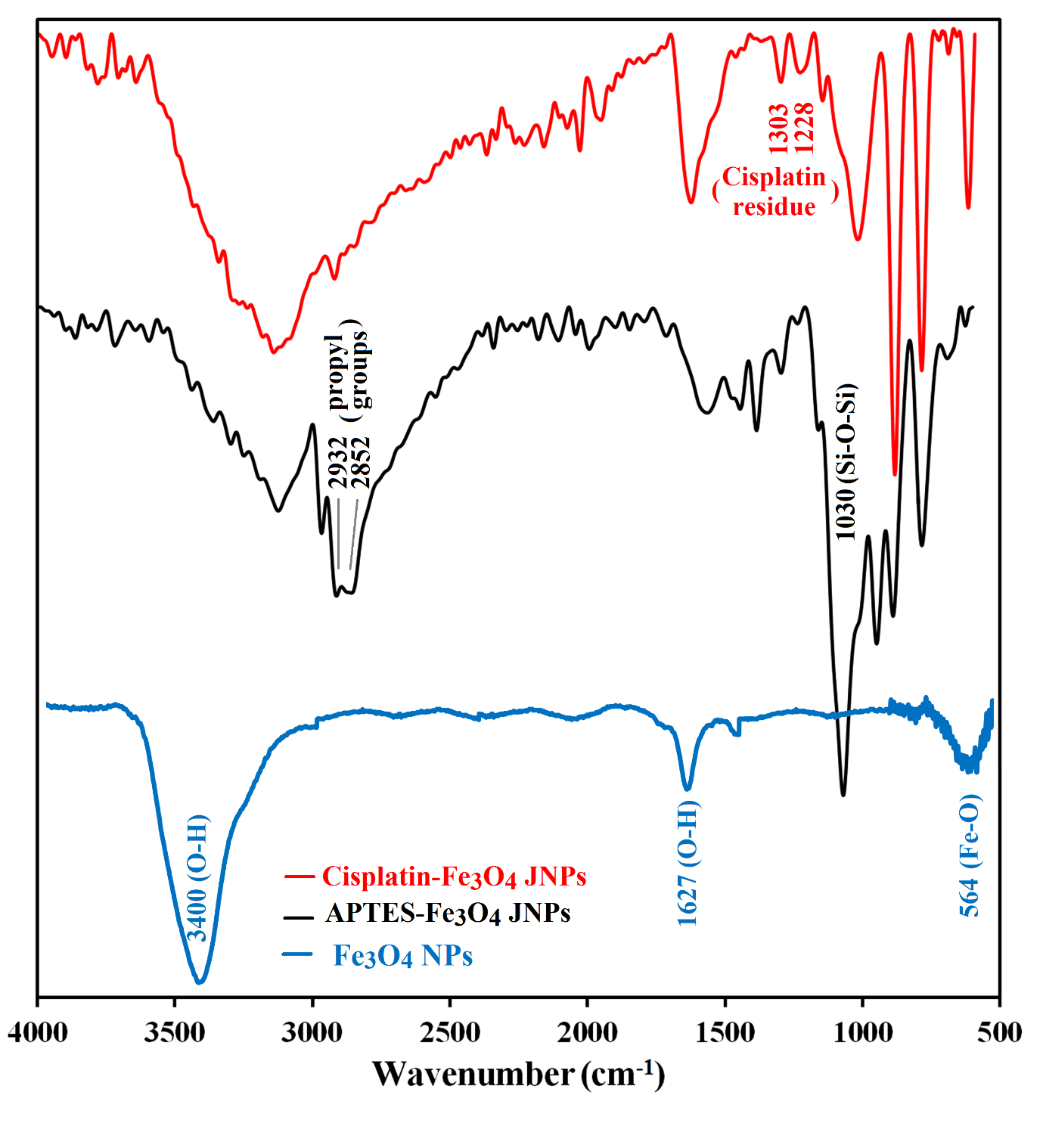


**Fig. S3.** FTIR spectra of Fe_3_O_4_ NPs (blue line), APTES-Fe_3_O_4_ JNPs (black line), and cisplatin-Fe_3_O_4_ JNPs (red line)


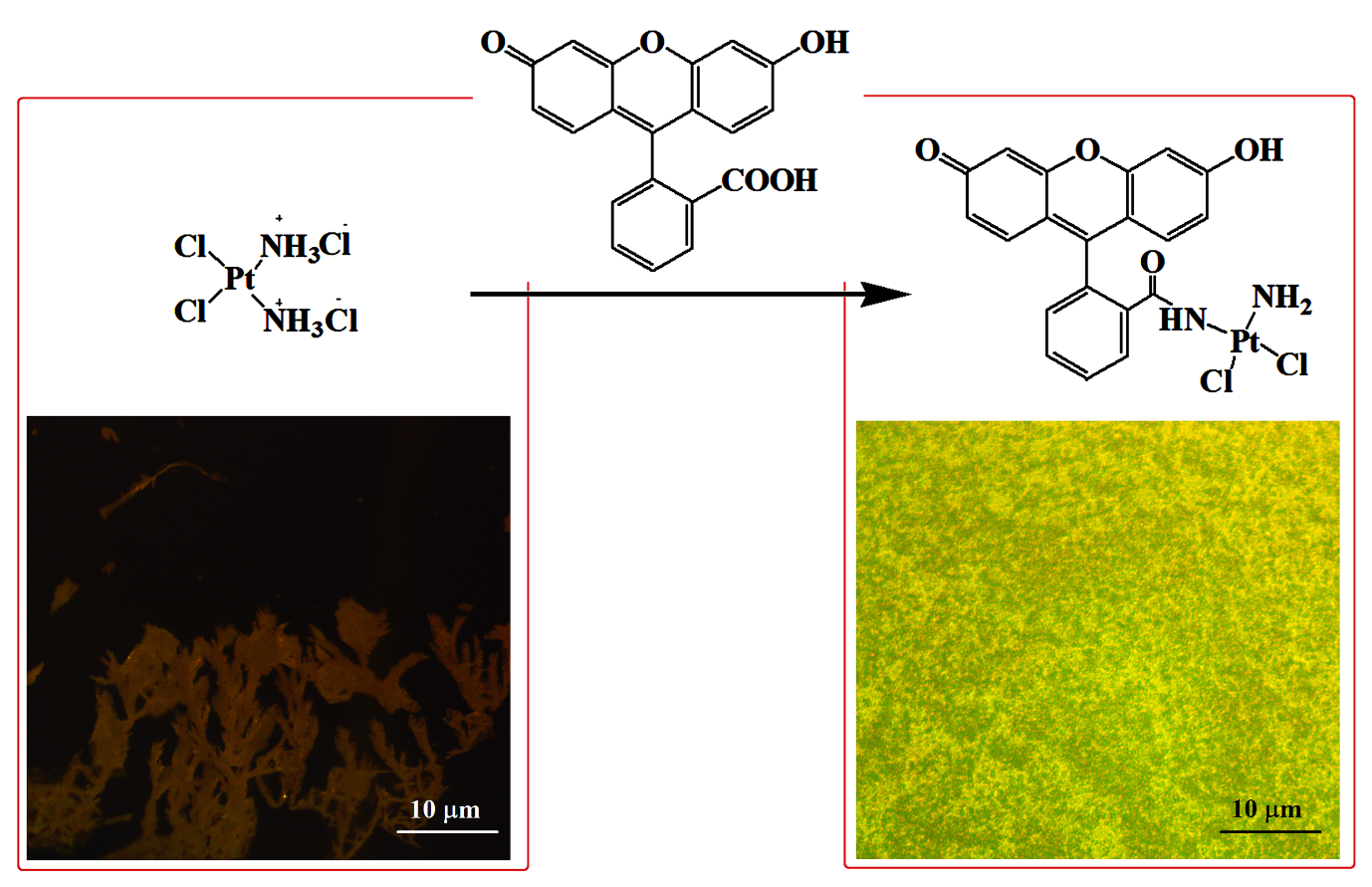


**Fig. S4.** The synthetic routes of untagged and fluorescein-labeled cisplatin (FL-CisPt) and their fluorescence micrographs


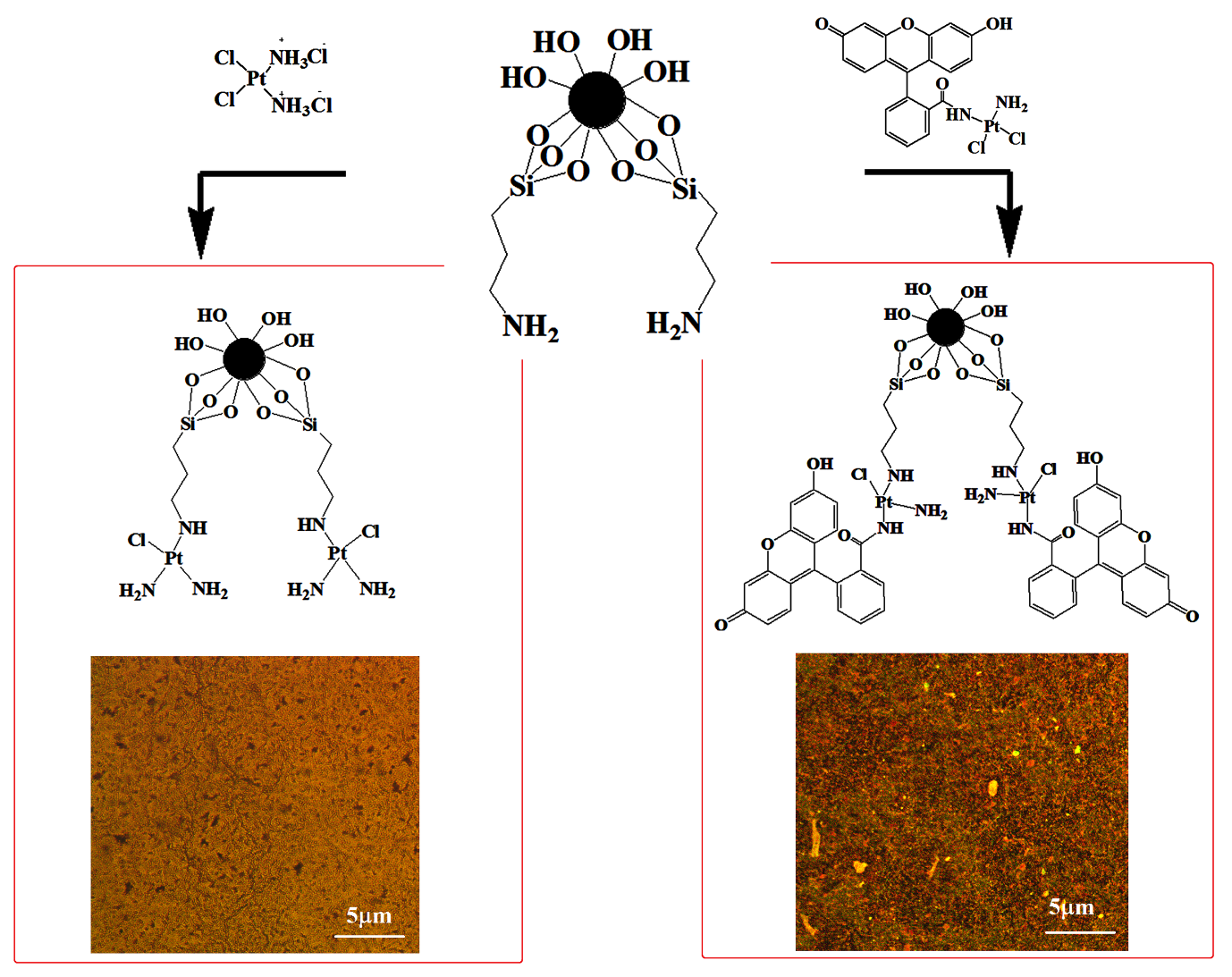


**Fig. S5.** The synthetic routes of untagged APTES-Fe_3_O_4_ JNPs and fluorescein-labeled APTES-Fe_3_O_4_ JNPs (FL-APTES-Fe_3_O_4_ JNPs) and their fluorescence micrographs


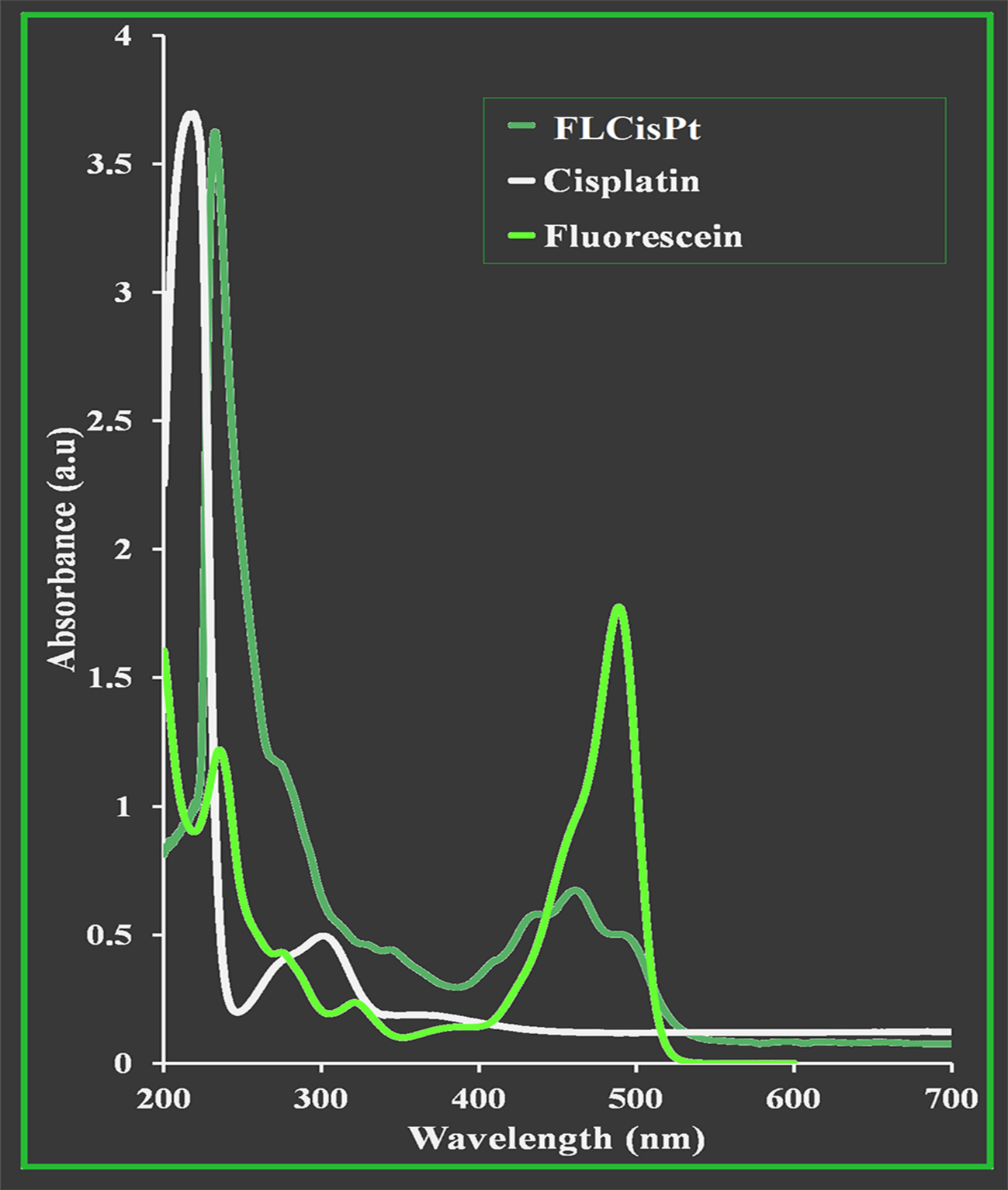


**Fig. S6.** UV-visible spectra of Cisplatin, Fluorescein, and FL-CisPt


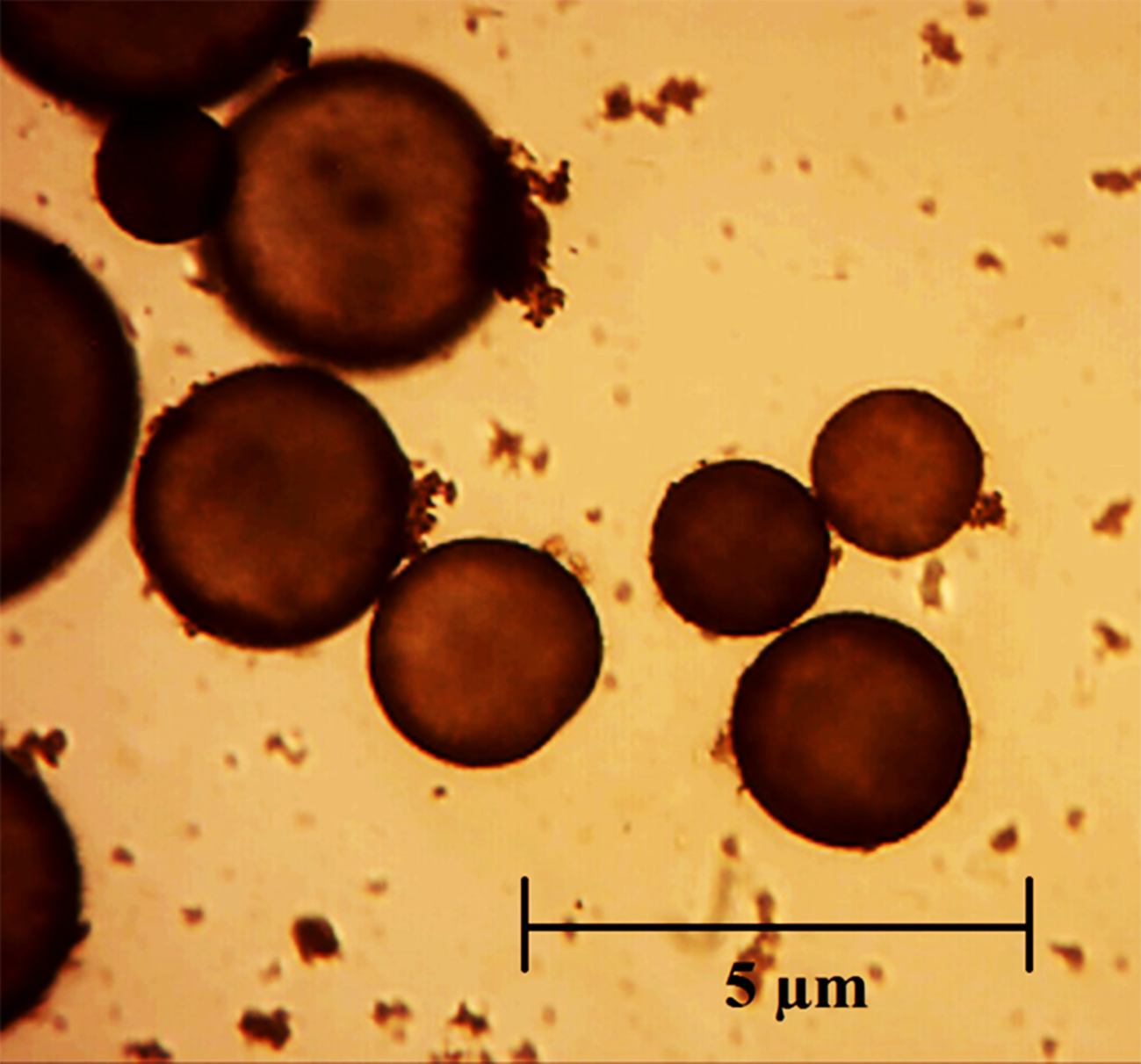


**Fig. S7.** Optical microscope image of CisPt-Fe_3_O_4_-wax microspheres with a typical particle size of 4.55 ± 1.43 µm (measured for N=100 particles)

**
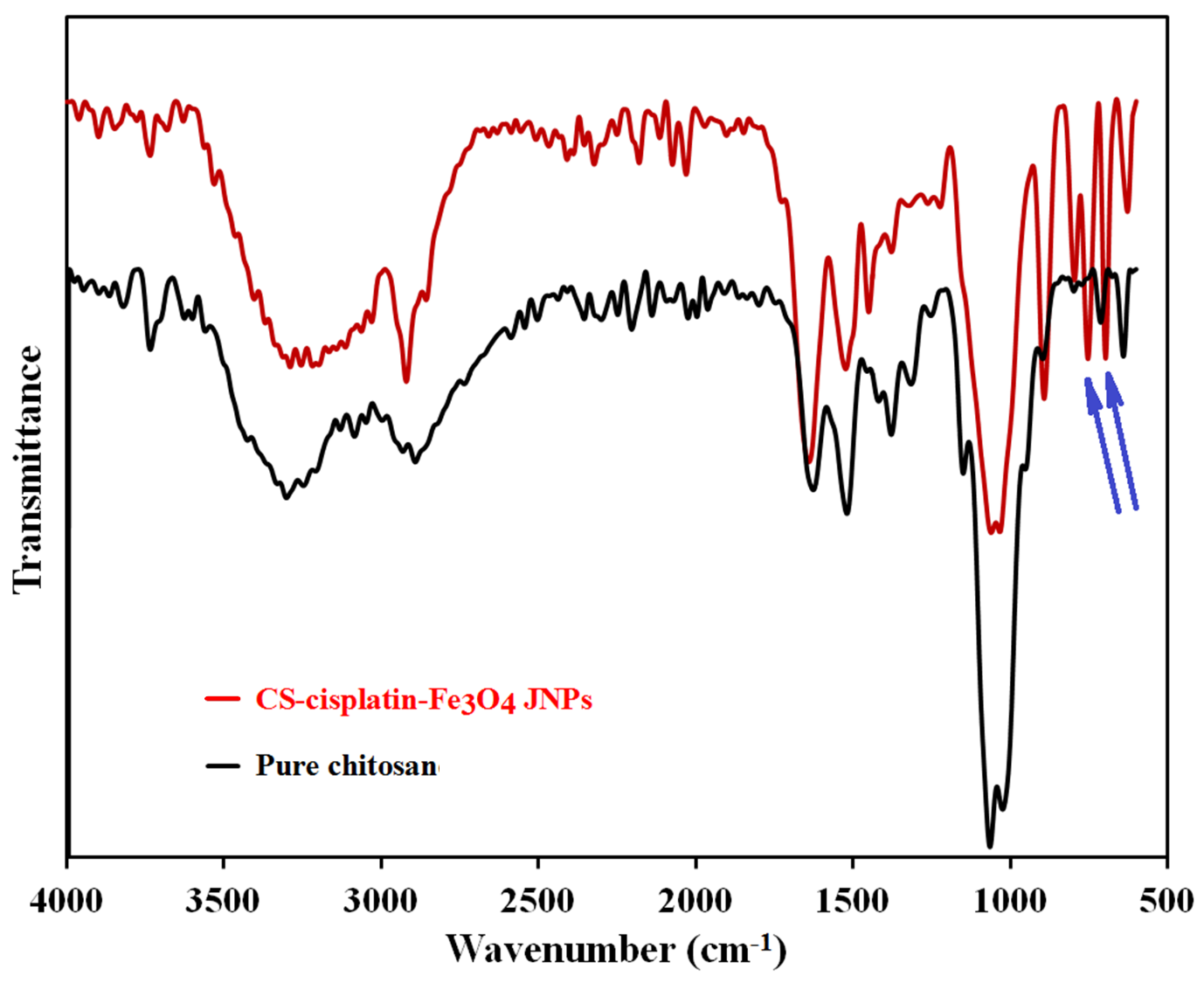
**

**Fig. S8.** Fourier-transform infrared (FTIR) spectrum of pure chitosan (black line) and Wax-free Cs-cisplatin-Fe_3_O_4_ JNPs (red line) (The arrows demonstrate the position of cisplatin chemical bonds)


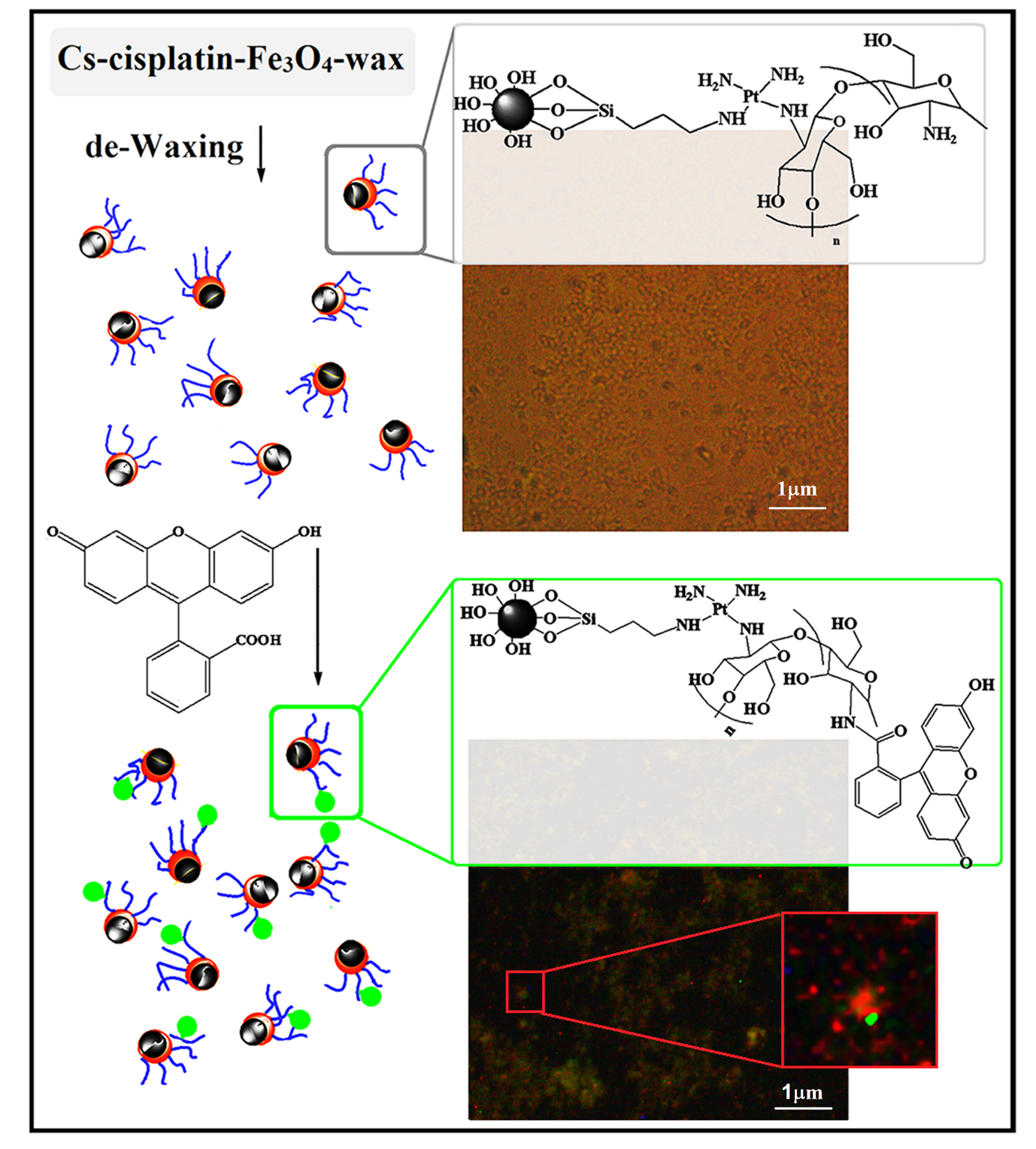


**Fig. S9.** The synthetic routes of untagged Cs-cisplatin-Fe_3_O_4_ JNPs and fluorescein-labeled Cs-cisplatin-Fe_3_O_4_ JNPs (FL-Cs-cisplatin-Fe_3_O_4_ JNPs) and their fluorescence micrographs

**
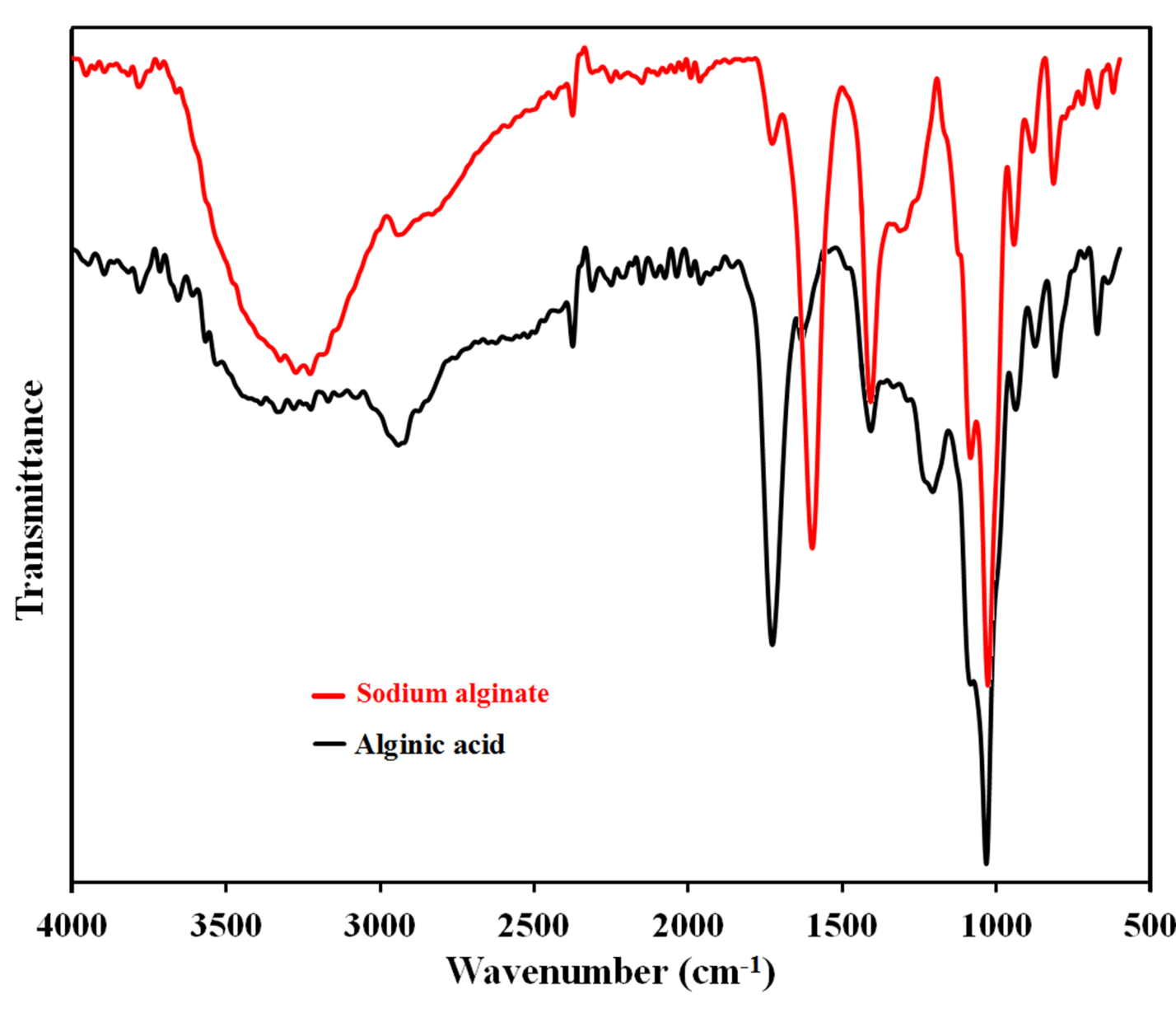
**

**Fig. S10.** FTIR spectrum of Sodium alginate (red line) and alginic acid (black line)
